# Personal Exposure to Source‐Specific Particulate Polycyclic Aromatic Hydrocarbons and Systemic Inflammation: A Cross‐Sectional Study of Urban‐Dwelling Older Adults in China

**DOI:** 10.1029/2023GH000933

**Published:** 2023-12-20

**Authors:** Jia Xu, Nan Zhang, Yujuan Zhang, Penghui Li, Jinbao Han, Shuang Gao, Xinhua Wang, Chunmei Geng, Wen Yang, Liwen Zhang, Bin Han, Zhipeng Bai

**Affiliations:** ^1^ State Key Laboratory of Environmental Criteria and Risk Assessment Chinese Research Academy of Environmental Sciences Beijing China; ^2^ Department of Family Planning The Second Hospital of Tianjin Medical University Tianjin China; ^3^ School of Environmental Science and Safety Engineering Tianjin University of Technology Tianjin China; ^4^ School of Quality and Technical Supervision Hebei University Baoding China; ^5^ School of Geographic and Environmental Sciences Tianjin Normal University Tianjin China; ^6^ Department of Occupational and Environmental Health School of Public Health Tianjin Medical University Tianjin China; ^7^ Tianjin Key Laboratory of Environment, Nutrition, and Public Health Tianjin Medical University Tianjin China; ^8^ Center for International Collaborative Research on Environment Nutrition and Public Health Tianjin China

**Keywords:** polycyclic aromatic hydrocarbons, inﬂammatory cytokines, source apportionment, cross‐sectional study, personal exposure, the elderly

## Abstract

Environmental exposure to ambient polycyclic aromatic hydrocarbons (PAHs) can disturb the immune response. However, the evidence on adverse health effects caused by exposure to PAHs emitted from specific sources among different vulnerable subpopulations is limited. In this cross‐sectional study, we aimed to evaluate whether exposure to source‐specific PAHs could increase systemic inflammation in older adults. The present study included community‐dwelling older adults and collected ﬁlter samples of personal exposure to PM_2.5_ during the winter of 2011. Blood samples were collected after the PM_2.5_ sample collection. We analyzed PM_2.5_ bound PAHs and serum inflammatory cytokines (interleukin (IL)1β, IL6, and tumor necrosis factor alpha levels. The Positive Matrix Factorization model was used to identify PAH sources. We used a linear regression model to assess the relative eﬀects of source‐specific PM_2.5_ bound PAHs on the levels of measured inflammatory cytokines. After controlling for confounders, exposure to PAHs emitted from biomass burning or diesel vehicle emission was significantly associated with increased serum inflammatory cytokines and systemic inflammation. These findings highlight the importance of considering exposure sources in epidemiological studies and controlling exposures to organic materials from specific sources.

## Key Points

- We evaluated the effects of source‐specific polycyclic aromatic hydrocarbons (PAHs) on inflammation in older adults
- Exposure to PAHs from biomass burning and diesel vehicles could increase specific cytokines
- This study highlighted the importance of source‐specific particulate matter in health studies

## 1 Introduction

As the products of incomplete combustion of organic materials (e.g., fossil fuel, biomass, etc.) (McGrath et al., 2007), polycyclic aromatic hydrocarbons (PAHs) have been believed to be one of the harmful constituents of atmospheric particulate matter (PM) (Y. Zhang & Tao, 2009). In toxicological studies, both in vivo and in vitro research have revealed the adverse effect of PM‐bound PAHs on the lungs via the pathway of inducing inflammation, oxidative stress, or cytotoxicity (He et al., 2017; Manzano‐León et al., 2016; Niu et al., 2017; Tong et al., 2019; Yang et al., 2017). As to the epidemiological evidence, several studies have proven that exposures to PM‐bound PAHs are associated with adverse health endpoints (Delfino et al., 2010a, 2010b; B. Feng et al., 2019; Shen et al., 2018; T. Wang et al., 2022; H. Xu et al., 2021).

Mechanistic studies reported that the inhalation of PAHs‐enriched PM_2.5_ (particulate matter with aerodynamic diameter equal to or less than 2.5 μm) can provoke reactive oxygen species (ROS) to induce local pulmonary and systemic inflammation (Medzhitov, 2008). After exposure to PM_2.5_ and associated PAHs, alveolar macrophages and bronchial epithelial cells secrete a growing amount of inflammatory biomarkers, including tumor necrosis factor‐α (TNFα), interleukin (IL) 1 beta (IL1β) and IL6 (Fujii et al., 2002; van EEDEN et al., 2001). Releasing these cytokines can activate a series of acute‐phase responses, which trigger systemic inﬂammation and consequent events (Dobreva et al., 2015; Medzhitov, 2008).

Several studies investigated the associations between systemic inflammation and PM constituents or sources (Altuwayjiri et al., 2021; Delfino et al., 2009; Hampel et al., 2015; Han et al., 2022; S. Wu et al., 2012). In these studies, exposure to constituents from vehicle emission (e.g., organic carbon (OC) and elemental carbon from tailpipe emission, Cu/Zn from non‐tailpipe emission) was found to enhance the levels of inflammatory cytokines. However, few studies linked PM_2.5_‐bound PAHs exposure to systemic inflammation and further investigated the emission sources that were more responsible for the pathophysiologic changes (Habre et al., 2018; T. Wang et al., 2022; H. Xu et al., 2021). Traditional source studies that applied the data of elements, water‐soluble ions, and carbonaceous fractions cannot distinguish specific sources with similar profiles, for example, emissions from gasoline and diesel vehicles. Apportioning PAHs by sources can determine these two sources (Larsen & Baker, 2003; Ravindra et al., 2008). In addition, as one of the essential parts of organic species, PAHs are believed to play a considerable part in the toxicological effects of PM_2.5_. Therefore, studies focusing on the associations between PM_2.5_‐bound PAHs and health endpoints are critical to elucidate the hazards of organic species and guide policy development. Moreover, previous studies used the concentrations of outdoor PM_2.5_‐bound PAH measured by monitors at fixed‐site as exposure metrics; however, they did not account for participant lifestyle or mobility or the differences between outdoor and indoor environments, which may result in exposure misclassification and biased assessment of the dose‐response relation (Avery et al., 2010). Applying exposure models or conducting personal sample collection for PM and constituents may help to minimize the impact of exposure bias (Mu et al., 2019).

Regarding the above research gaps, we conducted a cross‐sectional study to test the hypothesis that exposure to PAHs associated with PM_2.5_ can alter systemic inflammation by elevating cytokine levels and further identifying the sources responsible for the alteration. In this study, we measured the levels of personal exposure to PM_2.5_‐bound PAHs and those of serum inflammatory cytokines in older community‐dwelling adults in Tianjin, China. Source apportionment technology was applied to investigate the sources of PAHs and their contributions to increased levels of inﬂammatory cytokines. These findings may help explore the associations between source‐specific PAH exposure and systemic inflammation and may be used to guide policy development.

## 2 Materials and Methods

### 2.1 Study Participants and Design

This cross‐sectional study involved community‐dwelling adults (Figure S1 in Supporting Information S1) residing in Tianjin, China. In winter of 2011, 101 older adult volunteers were recruited and scheduled to collect personal exposure and fasting blood samples. An urban expressway with dense traffic is located 400 m away from the study community. The basic information of the study city was described in our previous publication (N. Zhang et al., 2022). We asked the staff of the local community center to help recruit the participants. The target population was the community‐dwelling older adults aged 65 to 80. During the recruitment, subjects were excluded if they could not take along the personal sampler, were in poor health, or had an activity range larger than 5 km from the residence. Before sample collection, all participants were requested to complete a person‐to‐person questionnaire and provide written informed consent for participation, as well as collect, store, and use their blood samples. The Institutional Review Board has approved the study for Human Subjects of Tianjin Medical University.

### 2.2 Sampling and Analyses

#### 2.2.1 Personal Exposure PM_2.5_ Sample Collection and PAHs Analysis

For each participant, the collection of PM_2.5_ personal exposure sample began at 8:00–9:00 in the morning of a given day and lasted for 24 hr during winter in 2011 (details are presented in our previous studies (Han et al., 2016; N. Zhang et al., 2022)). Briefly, we collected PM_2.5_ samples for personal exposure by requiring each participant to carry a backpack, in which we equipped two personal exposure monitors (PEM‐PM_2.5_; BGI, Inc., Waltham, MA, USA). We installed the inlets of the monitor on the shoulder straps of the backpack, making the inlets close to the breathing zone in all microenvironments. A 37 mm Teﬂon ﬁlter (Pall‐Gelman, Ann Arbor, MI, USA) and a 37 mm quartz ﬁlter (Pall‐Gelman, Ann Arbor, MI, USA) were equipped separately into the two samplers, which were connected by the pumps (Buck, Inc., Orlando, FL, USA), with a flow rate of 4 ± 0.2 L/min. Participants were required to always take along the backpack for 24 hr to obtain samples (approximately 5.76 m^3^ in total volume), except for sleeping, bathing, and clothing changes. They were allowed to place the backpack beside their bed during sleeping hours or nearby when resting for long periods, given the physical conditions of the older people. Due to the lack of samplers, participants underwent the sample collection on different days during the campaign. Finally, 87 valid 37‐mm quartz ﬁlter samples were collected for PAH analysis.

Gas chromatography coupled with mass spectrometry (GC‐MS, trace 2000 GC‐MS, Thermo Finnigan, Waltham, MA, USA) was used to analyze PAH samples, following previously described protocols (N. Zhang et al., 2022). The detailed information on the PAHs analysis is shown in Text S1, Tables S1, and S2 in Supporting Information S1. Meteorological parameters, such as relative humidity and temperature, were obtained from a local urban meteorological station.

#### 2.2.2 Source Apportionment for the PM_2.5_‐Bound PAHs

Source‐apportionment analysis for each participant was conducted using version 5.0 of the Environmental Protection Agency PMF model (Text S2 in Supporting Information S1). Source identification and contributions have been previously reported (N. Zhang et al., 2022), including two indoor sources (cooking fumes (CF) and environmental tobacco smoking (ETS)) and four outdoor sources (diesel vehicle emission (DV), coal combustion (CC), gasoline vehicle emission and biomass burning (BB)).

#### 2.2.3 Blood Collection and Analysis

After collecting PM_2.5_ personal samples, the participant was immediately escorted to the community health center for a blood draw and physical examination. Among 87 participants with valid personal PM samples, five did not provide blood samples for personal or medical reasons. Venous peripheral blood samples (5 mL) were collected by qualified physicians using coagulant vacuum tubes. We centrifuged the blood samples at 3,200 rpm for 10 min under room temperature and obtained the serum samples, which were then preserved at −80°C for further analysis. A Pro Human Inflammation Assays analytic kit (Bio‐Plex Assays, Bio‐Rad, Hercules, CA, USA) was used to measure cytokine levels. The target cytokines included IL1β, IL6, and TNFα, which have been proven to be associated with short‐term exposure to PM (Agrawal et al., 2018; Chen et al., 2015; Pope III et al., 2016; L. Tian et al., 2017; van EEDEN et al., 2001; Q. Zhang et al., 2020).

### 2.3 Statistical Analyses

Shapiro‐Wilk test showed that PAH exposure data were skewed (*P* < 0.05); thus, Spearman's rank correlation was used to check the relationships between PAH individuals. We log‐transformed the values of inflammatory cytokines. Multivariate linear regression was adopted to evaluate the associations of exposure to source‐specific PM_2.5_‐bound PAHs and inflammatory cytokine levels in participants. The introduction on how we carried out the contributions of each source using the linear regression models is listed in Text S3 in Supporting Information S1.

The contributions of each source for the participants, which were reported in our previous publication (N. Zhang et al., 2022), were separately accounted as independent variables in the linear regression models, while the inflammatory cytokines were dependent variables. All models were adjusted for confounders, including body mass index, sex, age, smoking status (active smoker, passive smoker, and non‐smoker), education (high school and below vs. college and above), relative humidity and outdoor temperature on the day of sample collection. To test if the smoking status modiﬁed the associations between exposure to source‐specific PAHs and systemic inflammation, we conducted stratified analysis in smokers (including active and passive smokers) and groups of participants that never smoked.

In addition, we conducted sensitivity analyses by (a) including the diagnoses of chronic diseases (e.g., hypertension, hyperlipidemia, diabetes mellitus) as covariates to minimize the possible influences of diseases that may have similar pathophysiologic pathways (T. Wang et al., 2022), (b) adjusting for the concentration of PM_2.5_‐bound OC to control for the association between OC and each source, (c) adjusting for PM_2.5_ mass concentration, (d) adjusting for the residual PM_2.5_ mass (residual PM_2.5_ mass concentration = PM_2.5_ mass concentration—sum of specific PAHs source mass), and (e) adjusting for the residual total PAHs mass concentration (residual total PAHs mass concentration = total PAHs mass concentration—sum of specific PAHs source mass).

## 3 Results

### 3.1 Basic Information of Participants

The participants' characteristics and inflammatory cytokine values are presented in Table 1. Eighty‐two participants were included in the analysis, with 47 women and 35 men and a median age of 66. Approximately 70% of participants are non‐smokers (neither active nor passive smokers), and one‐fifth of them received an education in college or higher. The levels of inflammatory cytokines varied among the participants. Normal distributions were observed among all log‐transformed inflammatory cytokine values (Shapiro‐Wilk test, *P* > 0.05). Table S3 in Supporting Information S1 further gives information on the physical examination of the participants. 45% of the participants have hypertension (systolic blood pressure ≥140 mmHg or diastolic blood pressure ≥90 mmHg at study examination time or a previous hypertension diagnosis by a physician), 33% of them have hyperlipidemia (total cholesterol >5.72 mmol/L or triglyceride >1.70 mmol/L at study examination time, or a previous hyperlipidemia diagnosis by a physician) (W. Feng et al., 2015), and 17% of them have diabetes (fasting glucose ≥7.0 mmol/L or a previous diabetes diagnosis by a physician).

**Table 1:** Participant Demographic Characteristics and Inflammatory Cytokine Levels (*N* = 82)

Variable	Median (5th, 95th percentiles) or percent
Age (years)	66 (60,77)
Sex	
Male	42.9%
Female	57.1%
Body mass index (kg/m^2^)	24.90 (20.23, 30.26)
Smoking	
Non‐smoker	67.9%
Passive smoker	12.5%
Active smoker	19.6%
Education level	
High school and below	79.5%
College and above	20.5%
IL1β (pg/mL)	5.00 (2.56, 8.66)
IL6 (pg/mL)	17.30 (8.86, 28.62)
TNFα (pg/mL)	52.30 (24.04, 141.19)

*Note*. IL, interleukin; TNFα, tumor necrosis factor alpha.

### 3.2 Characteristics of Source Contributions to PM_2.5_ Associated PAHs

Table 2 presents the concentrations and distributions of source contributions to total PAHs in this study. We have previously reported on personal exposure PAH levels distributions and source profiles identified in the PMF model (N. Zhang et al., 2022); this information is presented in Supporting Information S1 (Figures S2, S3, Texts S2, and S3).

**Table 2:** Summary Statistics of Source Contributions to Personal Exposure PM_2.5_‐Bound Polycyclic Aromatic Hydrocarbons (ng/m^3^)

Sources	Mean (SD)	Min	25th	Median	75th	Max	IQR
F1: Cooking Fumes (CF)	12.6 (12.7)	0.1	3.6	11.0	15.6	76.3	12.0
F2: Diesel Vehicle Emission (DV)	27.0 (23.1)	0.4	13.2	21.9	32.2	137.0	19.0
F3: Coal Combustion (CC)	23.7 (23.0)	1.0	12.9	18.6	28.9	156.1	16.0
F4: Environmental Tobacco Smoking (ETS)	11.3 (9.5)	0.2	4.8	8.7	14.4	56.1	9.6
F5: Gasoline Vehicle Emission (GV)	26.6 (14.9)	0.8	15.9	24.4	35.9	68.8	19.9
F6: Biomass Burning (BB)	22.7 (18.4)	0.6	11.3	17.0	26.9	93.4	15.6

*Note*. SD: standard deviation; IQR: interquartile range.

### 3.3 Associations Between Source‐Speciﬁc PAHs and Cytokines

Table 3 presents correlations among PAH sources. CC and CF, CC and diesel vehicle, CC and BB, as well as BB and ETS, had moderate correlations (*r* > 0.5); however, only the correlation between BB and ETS was statistically significant (*P* < 0.05), both of which are attributed to the combustion of plant residues.

**Table 3:** The Correlations Between Identified Sources

	CF	DV	CC	ETS	GV	BB
CF	1.00					
DV	0.35	1.00				
CC	**0.58**	**0.68**	1.00			
ETS	−0.03	0.44	0.27	1.00		
GV	0.06	−0.23	−0.01^a^	0.09	1.00	
BB	0.27	0.47	**0.50**	**0.58** ^a^	0.40	1.00

*Note*. CF: cooking fumes; DV: diesel vehicle emission; CC: coal combustion, ETS: environmental tobacco smoking; GV: gasoline vehicle emission; BB: biomass burning. Figures in bold indicate the correlation coefficient is higher than 0.5.

^a^ Represents *P* value < 0.05.

**Figure 1:**
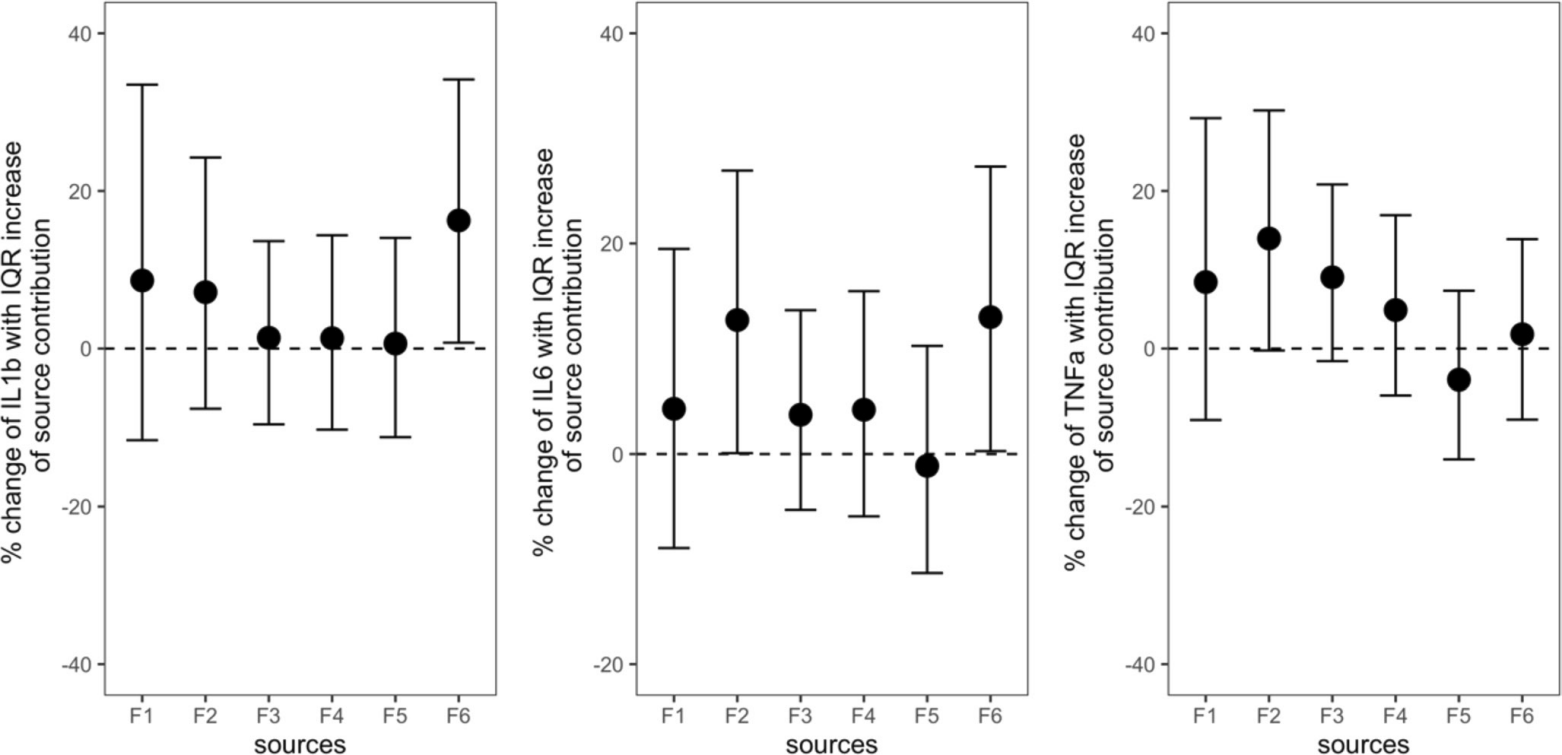
Estimates of IL1β, IL6 and tumor necrosis factor‐α (TNFα), with 95% confidence interval, per interquartile range increase of source specific polycyclic aromatic hydrocarbons concentrations analyzed by PMF (F1: Cooking Fumes, F2: Diesel Vehicle Emission (DV), F3: Coal Combustion, F4: Environmental Tobacco Smoking, F5: Gasoline Vehicle Emission, F6: Biomass Burning). Note: CI, confidence interval; IL, interleukin; TNFα, tumor necrosis alpha; IQR, interquartile range; PMF, Positive Matrix Factorization.

Figure S4 in Supporting Information S1 illustrates the associations between PAH individuals and cytokines. Only exposure to phenanthrene (PHE) was significantly associated with the increase of IL1β. Figure 1 shows the estimated changes of three inflammatory cytokines associated with exposure to source‐speciﬁc PAH concentrations. Positive effects were observed in most associations between cytokines and source‐specific PAHs, although some were not statistically significant. With an interquartile range (IQR) increase in the concentration of PAHs originated from biomass burning (15.6 ng/m^3^), the level of IL1β and IL6 were found to significantly increase (IL1β: 16.3%; 95% confidence interval: 0.7%, 29.8%; *P* < 0.05. IL6: 13.0%; 95% CI: 0.3%, 27.3%; *P* < 0.05). Exposure to PAHs originating from diesel vehicle emission was significantly associated with the increase in the level of IL6 (12.7%; 95% CI: 0.1%, 26.9%; *P* < 0.05), and marginally associated with an increase of TNFα levels (14.0%; 95% CI: −0.2%, 30.2%; 0.05 < *P* < 0.1). In addition, marginal association was observed between exposure to PAHs from CC and TNFα levels (9.0%; 95% CI: −1.6%, 20.8%; 0.05 < *P* < 0.1).

Stratiﬁed analyses showed that the associations between the concentrations of source‐specific PAHs and the levels of the cytokines varied considerably in different subgroups (Figure S5 and Table S4 in Supporting Information S1). The effects of biomass burning and diesel vehicle emission‐related PAHs are stronger in groups of participants that never smoked than in smokers (both active and passive smokers). In the subgroup of participants that never smoked, with an IQR increase in the PAHs originating from biomass burning, the levels of both IL1β and IL6 were observed to significantly increase (IL1β: 22.7%; 95% CI: 3.1%, 45.9%, *P* < 0.05. IL6: 19.5%; 95% CI: 3.3%, 38.1%; *P* < 0.05). Similarly, diesel vehicle emission‐related PAH exposure was marginally associated with levels of IL6 and TNFα (0.05 < *P* < 0.1). No significant associations were found in the subgroup of smokers (both active and passive).

As shown in Figure S6 in Supporting Information S1, the results remained robust after sensitivity analysis controlling for PM_2.5_‐bound OC, diagnoses, mass concentrations of PM_2.5_, residual PM_2.5_ mass concentration, or residual total PAHs.

## 4 Discussion

This cross‐sectional study with older adults as an object investigated the associations between personal exposure to source‐specific PM_2.5_‐bound PAHs and serum inflammatory cytokines. Our results found that exposure to most PAH sources could enhance the level of target cytokines and supported the hypothesis that exposure to certain source‐specific PM_2.5_‐bound PAHs can increase the markers of systemic inflammation among the elderly subpopulations. Specifically, exposure to PAHs emitted from biomass burning and diesel vehicle were significantly associated with serum levels of IL1β and IL6. These findings indicate that exposure to PAHs from different sources may differentially affect systemic inflammation.

This study highlighted the associations between serum inflammatory cytokines and exposure to PAHs from biomass burning or diesel vehicle emission. Previous studies reported the health effects of exposure to PM derived from these two sources. In California, exposure to PM_2.5_ emitted from biomass burning was found to increase the risk of mortality for all‐cause, cardiovascular diseases, and respiratory disease (Berger et al., 2018), as well as emergency department visits for respiratory disease (Ostro et al., 2016). A series of studies from New York State revealed that exposure to PM_2.5_ from biomass burning was associated with the enhancements of emergency department visits for asthma (Hopke et al., 2020) and hospitalizations for acute congestive heart failure (Rich et al., 2019). Meanwhile, PM_2.5_ from diesel exhaust could increase the risk of hospital admissions for neurodegenerative disease (van Wijngaarden et al., 2021) and acute ischemic heart disease (Rich et al., 2019). Few studies have examined the health effects caused by exposure to specific sources of PAHs. In a longitudinal follow‐up study in Beijing, B Feng et al. (2019) analyzed the multiple organic constituents of PM_2.5_ (including PAHs). They observed a significant arrhythmia risk increase associated with exposure to PM_2.5_ from coal burning, characterized by high concentrations of light PAHs (3‐ and 4‐ring PAHs). However, this study included multiple organic compounds and failed to isolate the effects of source‐specific PAHs. Two previous studies used the same PAHs data set and source contributions to investigate the associations between source‐specific PAHs and metabolic dysfunction and hemodynamic abnormality in healthy adults (H. Xu et al., 2021), as well as minor airway dysfunction in older adults with chronic obstructive pulmonary disease (T. Wang et al., 2022). Both studies reported that PM_2.5_‐bound PAHs from traffic emissions (including gasoline and diesel vehicle emissions) and coal burning were associated with increased related biomarkers. Similarly, our studies found that exposure to PAHs emitted from diesel vehicles increased the levels of inflammation markers. In addition, collecting personal samples allowed us to identify indoor PAH sources (e.g., CF, ETS, etc.) and examine their health effects. These indoor sources made marginal contributions to changes in inflammatory cytokine levels.

Interestingly, the associations between inflammatory cytokines and exposure to PAHs from biomass burning or diesel vehicle emission were stronger among the participants who never smoked (neither active nor passive smokers). One reasonable explanation is that smoking is supposed to bring a higher effect on systemic inflammation than PM‐bound PAHs and, therefore, partially masks the effects of PAHs. Nevertheless, the joint effects of PM_2.5_ constituents and ETS on systemic inflammation are still under‐researched. More studies are required to explore the interactions between PM_2.5_ constituents (e.g., heavy metal, PAHs, etc.) and cigarette smoking on respiratory health (Mu et al., 2021).

Toxicological study has revealed that exposure to PAHs can trigger a strong proinﬂammatory response, in which macrophages release an increasing quantity of proinﬂammatory cytokines IL1β, IL6, and TNFα (Pardo et al., 2020). Experimental studies provided substantial toxicological evidence that PAHs from biomass burning or diesel vehicle emission can increase the levels of these proinﬂammatory cytokines. Jiang et al. (2019) found that organic compounds from biomass burning (including PAHs) increase the levels of IL1β, IL6, and TNFα. An in vitro study (F. Xu et al., 2020) reported that vehicle emissions exhibited significant positive correlations with the levels of TNFα and ROS. In addition, the study found that a one‐fold increase in vehicle‐emitted compositions could lead to a 7.5% increase in ROS production rate and a 13.0% increase in TNF‐α levels. In a study from Tehran (Iran), diesel vehicle emissions were associated with TNF‐α levels in cold seasons, suggesting a critical role of organic compounds (e.g., PAHs) in inflammation or oxidative stress (Al Hanai et al., 2019). A Hong Kong study revealed that PAHs emitted from high‐duty diesel vehicles triggered the secretion of larger quantities of IL6 than those emitted by other vehicle types (B. Wang et al., 2021).

It is worth noting that the work presented herein was conducted in 2011, which was conducted over a decade ago. Changes in external factors, such as energy structures and living conditions, may have affected the current results. Consequently, it is crucial to juxtapose the current findings with more recent studies. To be specific to the source apportionment results, there have been no publications related to the source analyses on PM_2.5_ bound PAHs in the study city over the past decade. Only three studies have reported on the levels of PM_2.5_ bound PAHs in the ambient atmosphere (L. Feng et al., 2015; C. Wang & Cui, 2020; Y. Wang et al., 2019). These data allow for a longitudinal comparison with our study on the levels of total PAHs (Table 4). Additionally, the ratios between certain PAHs can help distinguish their sources, so we have selected three ratios for comparison (Table 4). Although the levels of total PAHs varied evidently, the values of the three ratios from this study compared with the other three studies are similar. Therefore, even though this study was conducted over a decade ago, the relative contribution of each source to the total PAHs has not noticeably changed. Moreover, we also considered other studies conducted in the study city concerning source apportionment on PM_2.5_ (Gao et al., 2018; Huang et al., 2017; Liu et al., 2020; Paatero, 1997; Paatero & Tapper, 1994; Shi et al., 2018; Y. Tian et al., 2016, 2018; Wen et al., 2018; H. Wu et al., 2015; Xiao et al., 2020; Yuan et al., 2018; W. Zhang et al., 2021). The difference is that these studies focused on the compositions of PM_2.5_ bound elements, water‐soluble ions, and carbonaceous fractions rather than PAHs. The results of these studies, shown in Table S5 in Supporting Information S1, also indicate minor changes in the relative contributions of each source to the PM_2.5_ mass over the years. In addition, the main form of biomass burning in China is crop straw burning (Zhao et al., 2022), which mostly happens in rural areas. Therefore, the biomass burning related PAHs identified in this study are supposed to originate from nearby rural areas and be transmitted to the urban area through atmospheric transportation.

**Table 4:** The Comparisons on the Ratios of Certain Polycyclic Aromatic Hydrocarbon Individuals in Different Studies Conducted in Tianjin

Data source	Sampling period	Total PAHs (ng/m^3^)	IND/(IND + BghiP)	BaA/(BaA + CHR)	FLU/(FLU + PYR)
This study	December 2011	112.3	0.56	0.41	0.56
L. Feng et al. (2015)	December 2013–January 2014	180.93	0.5	0.37	0.69
C. Wang and Cui (2020)	October 2016–March 2017	69.29	0.53	0.46	0.48
C. Wang and Cui (2020)	October 2017–March 2018	33.27	0.5	0.42	0.51
Y Wang et al. (2019)	March 2017–February 2018	46.1	0.51	0.43	0.53

Our study has several strengths. First, the personal exposure sampling strategy allowed us to estimate the accurate exposure to evaluate the impact of indoor exposures that the participants underwent and to identify the information on indoor sources (e.g., CF, ETS). Second, source‐specific PM_2.5_‐bound PAHs were evaluated. These results may support source control and policy development.

However, we admit that the following limitations restrict the interpretation of the results. First, the impact of exposure to air pollution on inflammatory markers was evaluated approximately concurrently (Q. Zhang et al., 2020). The PAH levels were obtained 24 hr before the biomarkers were measured; samples on days two and three post‐exposure were not evaluated. This approach may have underestimated any potential lag effects. Second, this study analyzed only PM_2.5_‐bound PAHs. At the same time, the possible effects of other unmeasured organics, such as PAHs derivatives (e.g., quinones and nitrated PAHs), secondary organic aerosol, and other compounds (F. Xu et al., 2020), were not considered. Third, our study recruited only older adults, limiting the generalizability of the results, which is applicable to only a tiny portion of the population. Future studies should evaluate other populations, including children, infants, pregnant women, etc., to achieve more comprehensive evidence for policy development. Fourth, the cross‐sectional study design does not help determine cause and effect, and the snapshot monitor on PM_2.5_ and cytokines may provide differing results if another time frame had been chosen. Fifth, this study was conducted in 2011, which has been more than 10 years from now. The energy structure, living standards, habits, and other factors have greatly varied over the years. Therefore, new studies were required to better characterize the present exposure and health outcomes.

## 5 Conclusions

In conclusion, this study evaluated the associations between exposure to source‐specific PAHs and inflammatory cytokine levels to elucidate the influences of emissions sources on human health. Exposure to PAHs emitted from biomass burning or diesel vehicle emission may increase serum cytokine levels and enhance systemic inflammation. These results illustrate the health effects of PAH emission sources, suggesting controlling air pollution may help reduce human health risks. The study implies that estimating the health effects at the level of emission source is capable of promoting policy development, such as targeting sources that are harmful to human health and minimizing possible exposure to these sources.

## Conflict of Interest

The authors declare no conflicts of interest relevant to this study.

## Supporting information

Supporting Information S1

## Data Availability

Data is available at Han (2023). Figures were made with R (version 4.1.1; R Project for Statistical Computing).
