# Supplementary material for: Personal Exposure to Source‐Specific Particulate Polycyclic Aromatic Hydrocarbons and Systemic Inflammation: A Cross‐Sectional Study of Urban‐Dwelling Older Adults in China
Source: Geohealth. 2023 Dec 20;7(12):e2023GH000933. doi: 10.1029/2023GH000933 (PMC10731620; doi:10.1029/2023GH000933)
Supplement: Supplementary file 1 — Supporting Information S1 [file GH2-7-e2023GH000933-s001.docx]

**Supporting Information for**

**Personal exposure to source-specific particulate polycyclic aromatic hydrocarbons and systemic inflammation: A cross-sectional study of urban-dwelling older adults in China**

Jia Xu^1#^, Nan Zhang^1#^, Yujuan Zhang^3,1^, Penghui Li^4^, Jinbao Han^5^, Shuang Gao^6^, Xinhua Wang^1*^, Chunmei Geng^1^, Wen Yang^1^, Liwen Zhang^2,7,8^, Bin Han^1^*, Zhipeng Bai^1^

1. State Key Laboratory of Environmental Criteria and Risk Assessment, Chinese Research Academy of Environmental Sciences, Beijing 100012, China

2. Department of Occupational and Environmental Health, School of Public Health, Tianjin Medical University, Tianjin 300070, China

3. Department of Family Planning, The Second Hospital of Tianjin Medical University, Tianjin 300211, China

4. School of Environmental Science and Safety Engineering, Tianjin University of Technology, Tianjin 300384, China

5. School of Quality and Technical Supervision, Hebei University, Baoding 071002, China

6. School of Geographic and Environmental Sciences, Tianjin Normal University, Tianjin 300387, China

7. Tianjin Key Laboratory of Environment, Nutrition, and Public Health, Tianjin Medical University, Tianjin, 300070, China

8. Center for International Collaborative Research on Environment, Nutrition and Public Health, Tianjin, 300070, China

# These two authors contributed equally to the paper.

*Corresponding authors: Bin Han (hanbin@craes.org.cn) and Xinhua Wang (wangxh@craes.org.cn)

**Table of contents**

Text S1 Laboratory analysis for PAHs

Text S2: Introduction of the Positive Matrix Factorization (PMF) model

Text S3 The formula of the linear regression

Figure S1 Location of the research community

Figure S2 Source profile obtained from PMF model (the bars represent the percentage of the constituent of the specific source in the total observed mass of the constituent)

Figure S3 Comparisons between measured and modeled PAH individuals

Figure S4 Associations between source-specific PAHs and cytokines, stratiﬁed by smoking status

Figure S5 Associations between source-specific PAHs and cytokines, stratiﬁed by smoking status

Figure S6 The estimates with 95%CI of inflammatory cytokines with per IQR increase of source-specific PM_2.5_ concentrations before and after controlling for OC, disease conditions, PM_2.5_ mass concentration, residual PM_2.5_ mass, concentration, or residual total PAHs mass concentration

Table S1 Summary of the PAH analysis and Quality Control/Quality Assurance

Table S2 PAHs concentration in standard solution grads (μg/ml)

Table S3 Basic characteristics and clinical parameters of participants (N=82)

Table S4 Estimated changes of cytokines with IQR increase of source-specific PAHs concentrations analyzed by PMF exposure stratiﬁed by smoking status

Table S5 The source apportionment results in the study city at different years

# Text S1 Laboratory analysis for PAHs

Before sampling, all blank filters were conditioned in our controlled balance lab (21±2 ^o^C and 40±5% relative humidity) for at least 48 h. Collected filters were kept in sampling petri dishes and stored in the refrigerator below -20°C before analysis.

The filter samples were extracted with dichloromethane by ultrasonication three times (20 min each time) in 150 mL of dichloromethane. After extraction, rotary evaporation was used to concentrate the extracts in a final volume of approximately 5 mL, and then, the extracts were concentrated under a gentle stream of 99.999% pure nitrogen gas flow, until 1 mL was obtained.

After each sample was transferred onto a separation column, a mixed solution of n-hexane and dichloromethane (1:1) was added to eliminate impurities. Finally, all purified extracts were reduced to 1.0 mL under a gentle stream of 99.999% pure nitrogen gas flow.

A gas chromatograph was connected to a mass spectrometer (model 6890N-5973i, Agilent, USA) and an Agilent ChemStation data system to determine these twelve PAH species. The compounds were separated on a fused silica capillary column (30m×0.3mm×0.25μm). The injection temperature was 285°C. The oven temperature was 70°C for 4 min and was increased to 300°C at 10 °C/min. This was held for 2 min, then increased to 340°C at 5 °C/min, then held for 12 min. In this experiment, helium was used as the carrier gas.  The MS source temperature was 230 °C. Target PAHs were identified based on the standards' retention time and qualitative ions in a selected ion monitoring mode that were then quantified by the internal standards (Table S1). The internal standards used in this study were: D_12_-Perylene for BeP, BaP and BkF, D_12_-Chrysene for BaA, CHR and PYR, D_10_-Acenaphthene for FLU, BghiP, IND, BbF and COR, D_10_-Phenanthrene for PHE.

Standard reference materials used in this study were the mixture of EPA 610 (Polynuclear Aromatic Hydrocarbons Mix, Supelco #48743, containing 16 PAH individuals), BeP standard (Supelco #36962), and Cor Standard (Supelco #36963). The range of standard solutions is shown in Table S2.

# Text S2 Introduction of the Positive Matrix Factorization (PMF) model

PMF model was developed by Paatero [[*Paatero*, 1997](#_ENREF_4); [*Paatero and Tapper*, 1994](#_ENREF_5)] and has been a widely applied model with unknown source profiles, especially after being released by the U.S. EPA. In this model, a data matrix X, in which *i* is the samples and *j* is the chemical species measured, can be viewed as a speciated data set, and the concept of this model is shown in Equation (1):

$X_{ij}=\sum_{k=1}^{p} g_{ik}f_{kj}+e_{ij}$ (1)

where $p$ is the number of factors; $f$ is the chemical profile of each source, $g$ is the mass contribution by each factor to each sample; $f_{jk}$ is the source profile, $e_{ij}$is the residual for each species or sample.

PMF solves equation (1) by minimizing the sum of the square of residuals weighted inversely with error estimates of the data points, $Q$, which is defined as

$Q=\sum_{i=1}^{n} \sum_{j=1}^{m} \left[ \frac{x_{ij}-\sum_{k=1}^{p} g_{ik}f_{kj}}{u_{ij}} \right]^{2}$ (2)

Where$u_{ij}$ is the uncertainty of each species in the model, and it can be calculated using the following equation:

$Uncertainty=\sqrt{{(Error Fraction \times concentrion)}^{2}+{(MDL)}^{2}}$ (3)

Where: MDL here is the method detection limit of each constituent.

# Text S3 The formula of the linear regression

The formula of the linear regression is shown in Eq (1)

g(μ) = β_0_ + β_1_(source contribution) + [γ’X] (1)

The model is represented in the form of a linear regression model, with the dependent variable for the levels of inflammatory factors (μ) expressed as a function of the independent variables for the contributions of specific sources to total PAHs and a matrix of other covariates[γ’X].

Linear regression coefficients (β) and 95% confidence intervals (CIs) of the model were interpreted as percent changes, calculated as (e^β^ - 1) × 100. Model results are expressed about each interquartile range (IQR) increase in the contribution of specific sources to total PAHs.

# Figure S1 Location of the research community


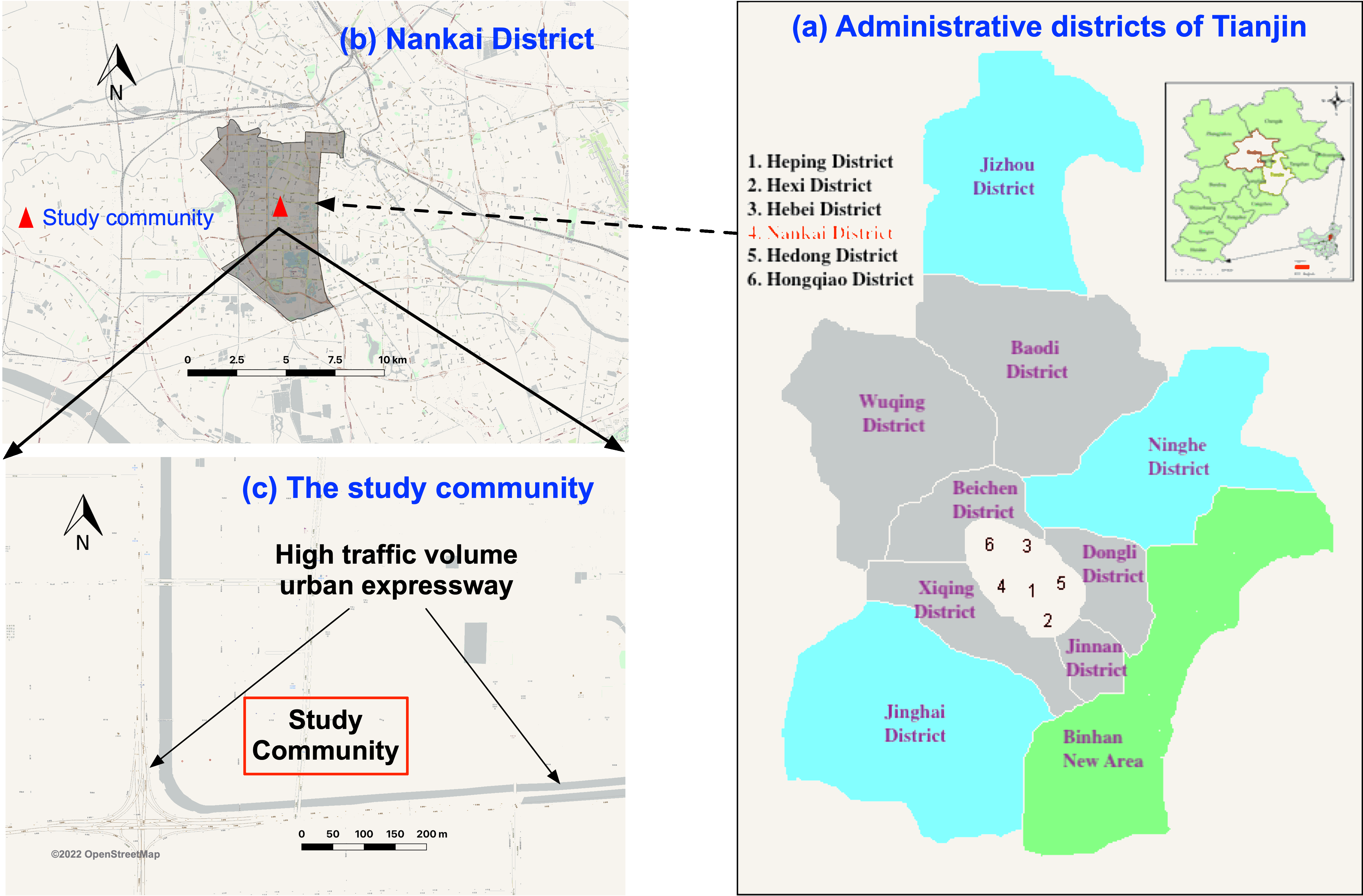


Figure S1 Location of the research community (a. Administrative district of Tianjin; b. the district where the study community is located; c. the study community)

(Map sources: a. https://www.travel-zentech.jp/English/Map/China/Tianjin.htm; b. OpenStreetMap; c. OpenStreetMap)

# Figure S2 Source profile obtained from PMF model (the bars represent the percentage of the constituent of the specific source in the total observed mass of the constituent)

Fig.1. Source profile of personal PM_2.5_-bound PAHs exposure.

Figure S2 Source profile obtained from PMF model (the bars represent the percentage of the constituent of the specific source in the total observed mass of the constituent)

Note: (F1: Cooking Fumes, F2: Diesel Vehicle Emission, F3: Coal Combustion, F4: Environmental Tobacco Smoking, F5: Gasoline Vehicle Emission, F6: Biomass Burning)

# Figure S3 Comparisons between measured and modeled PAH individuals

*
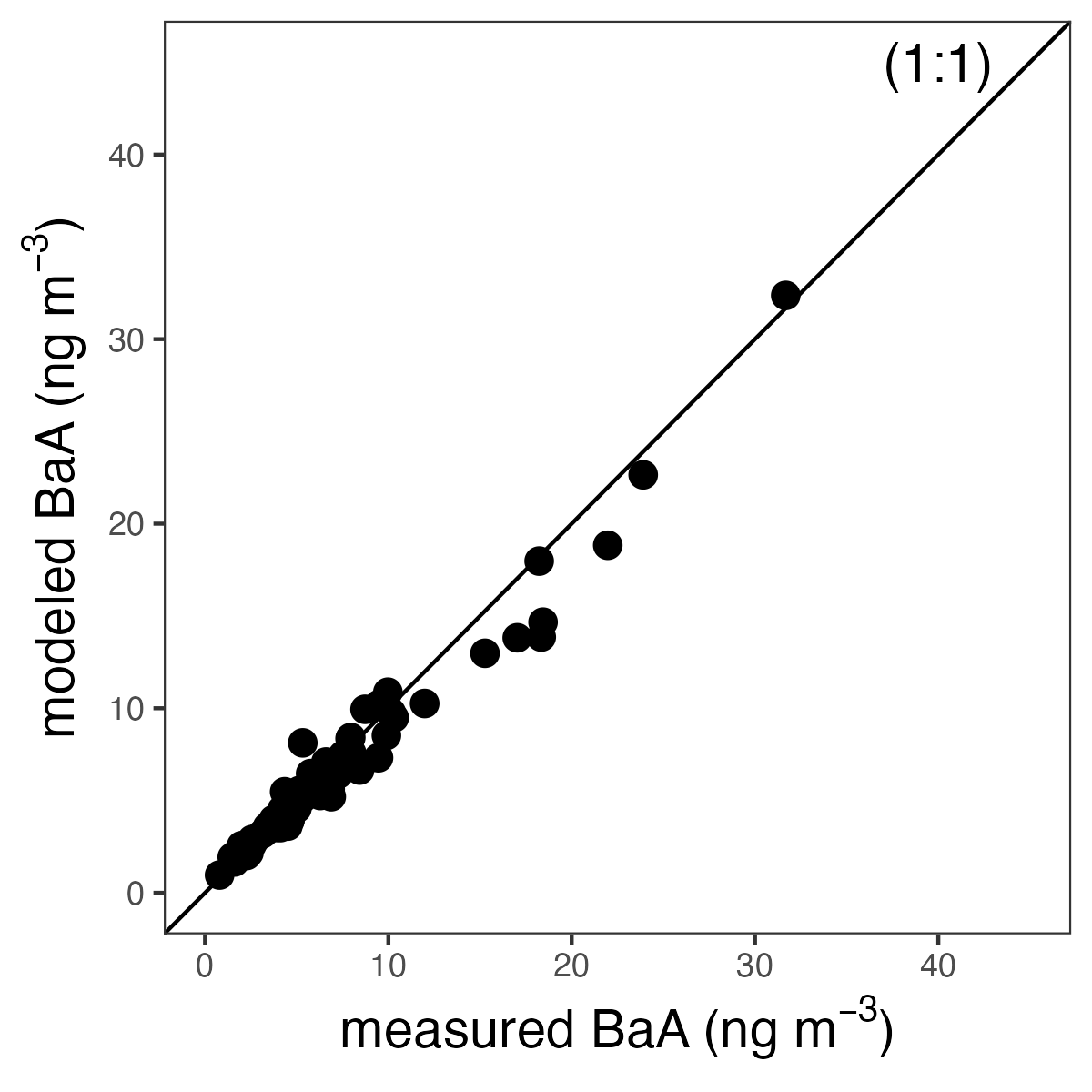

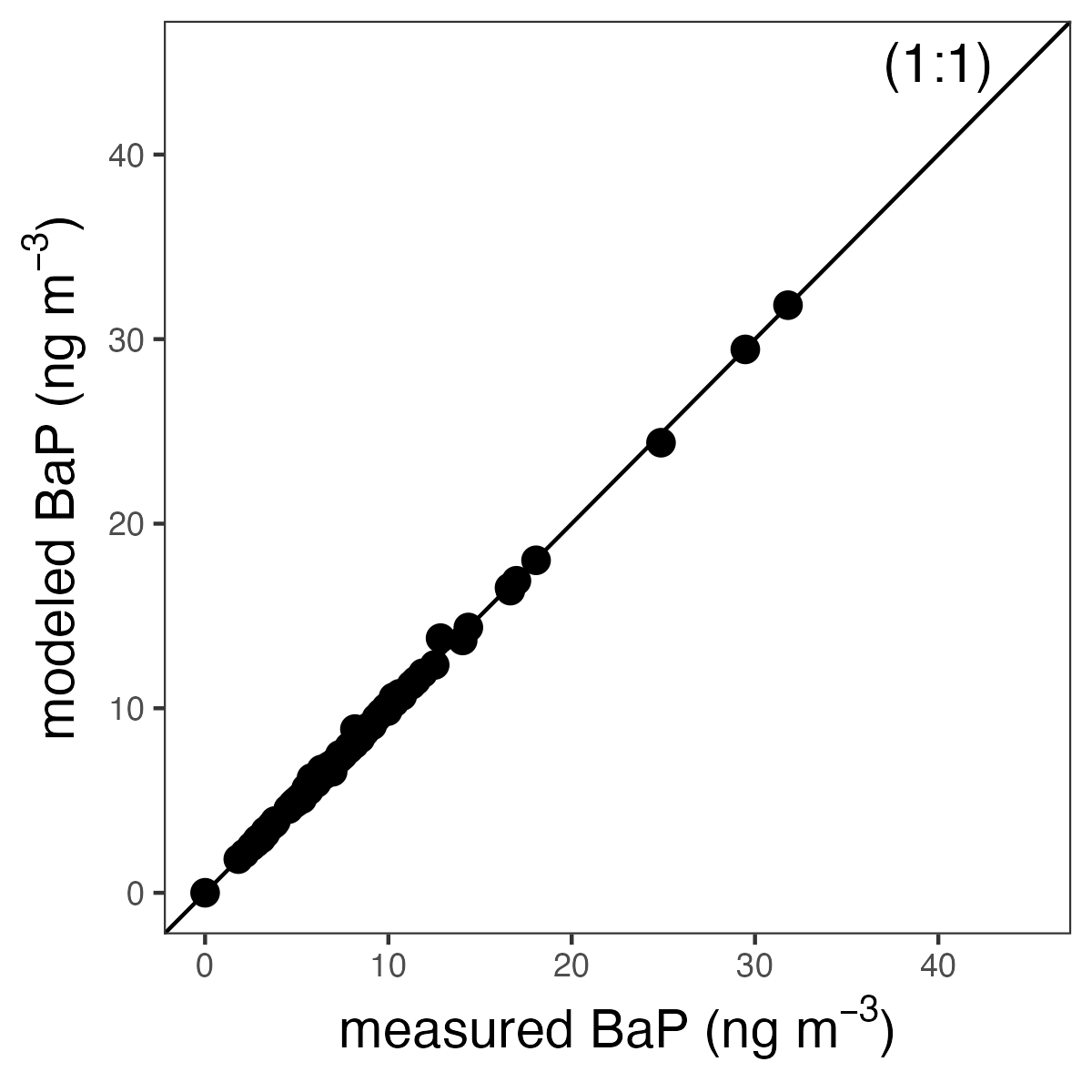

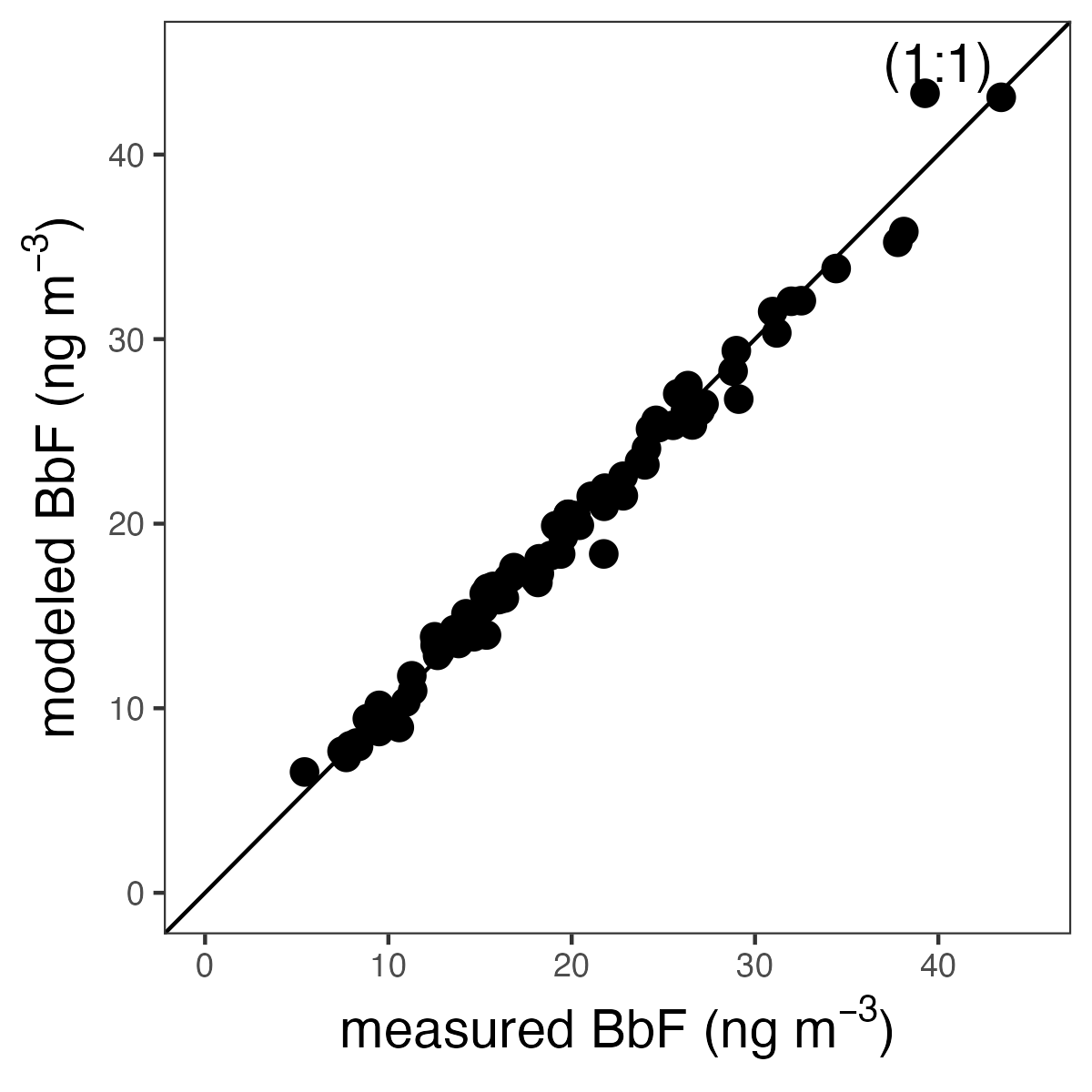

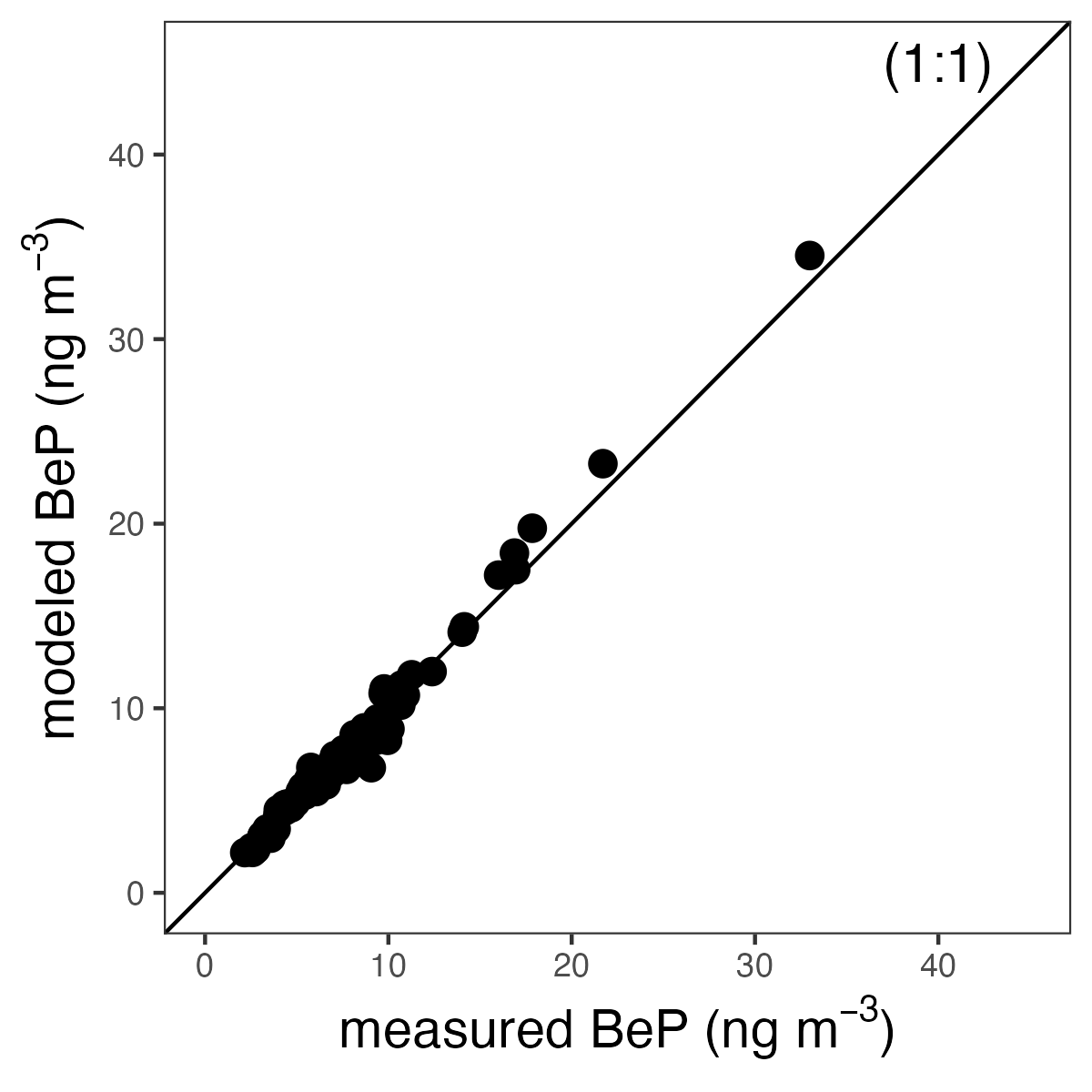

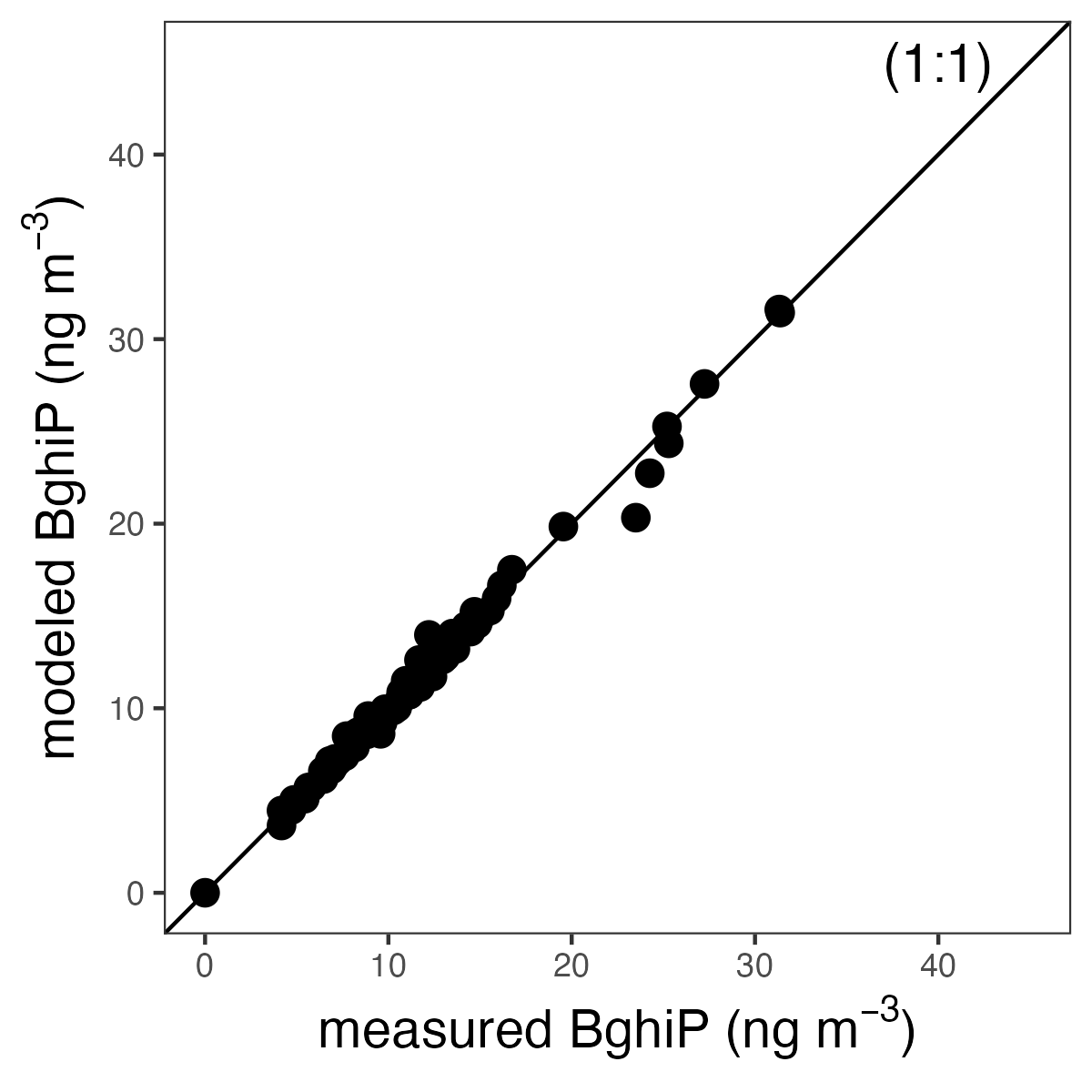

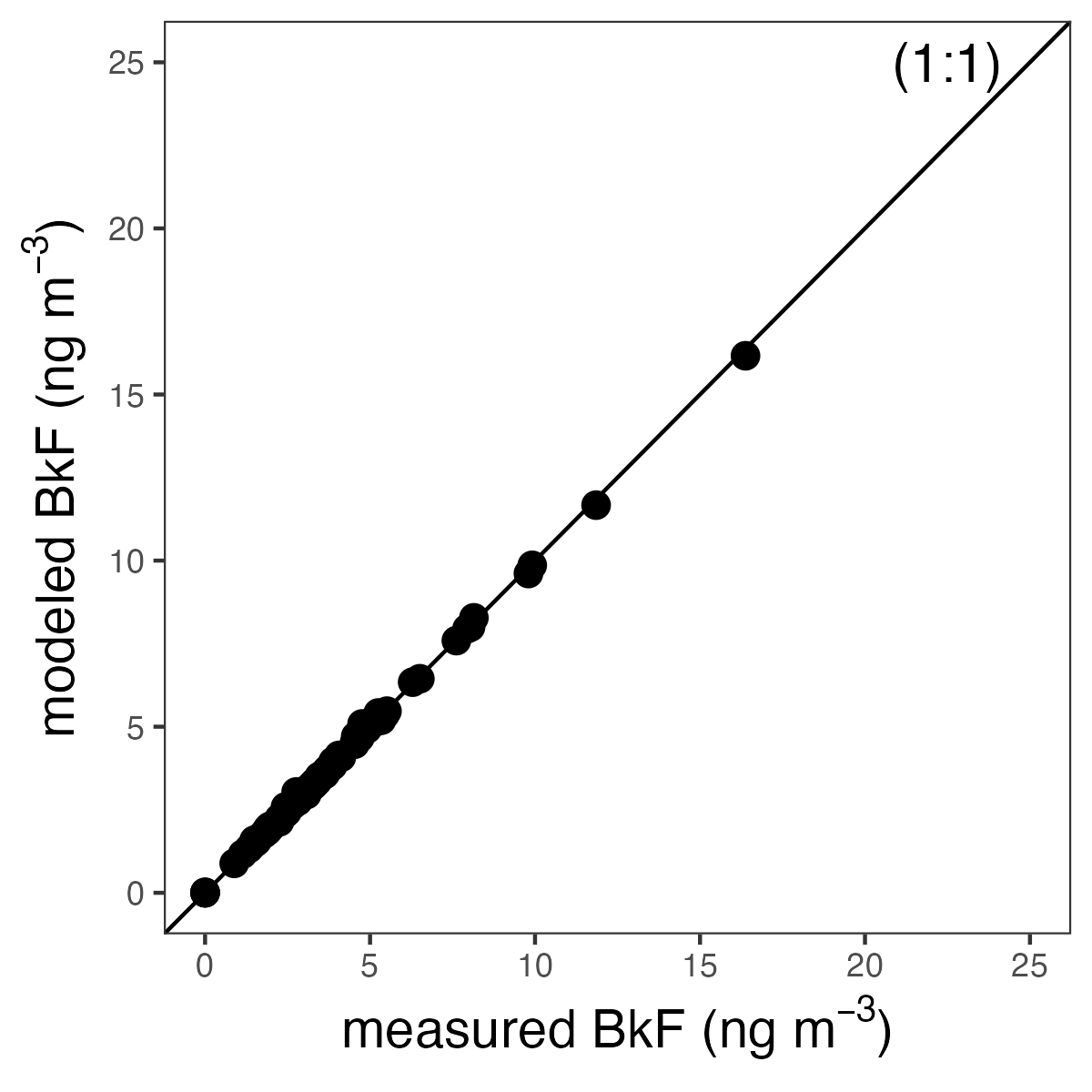

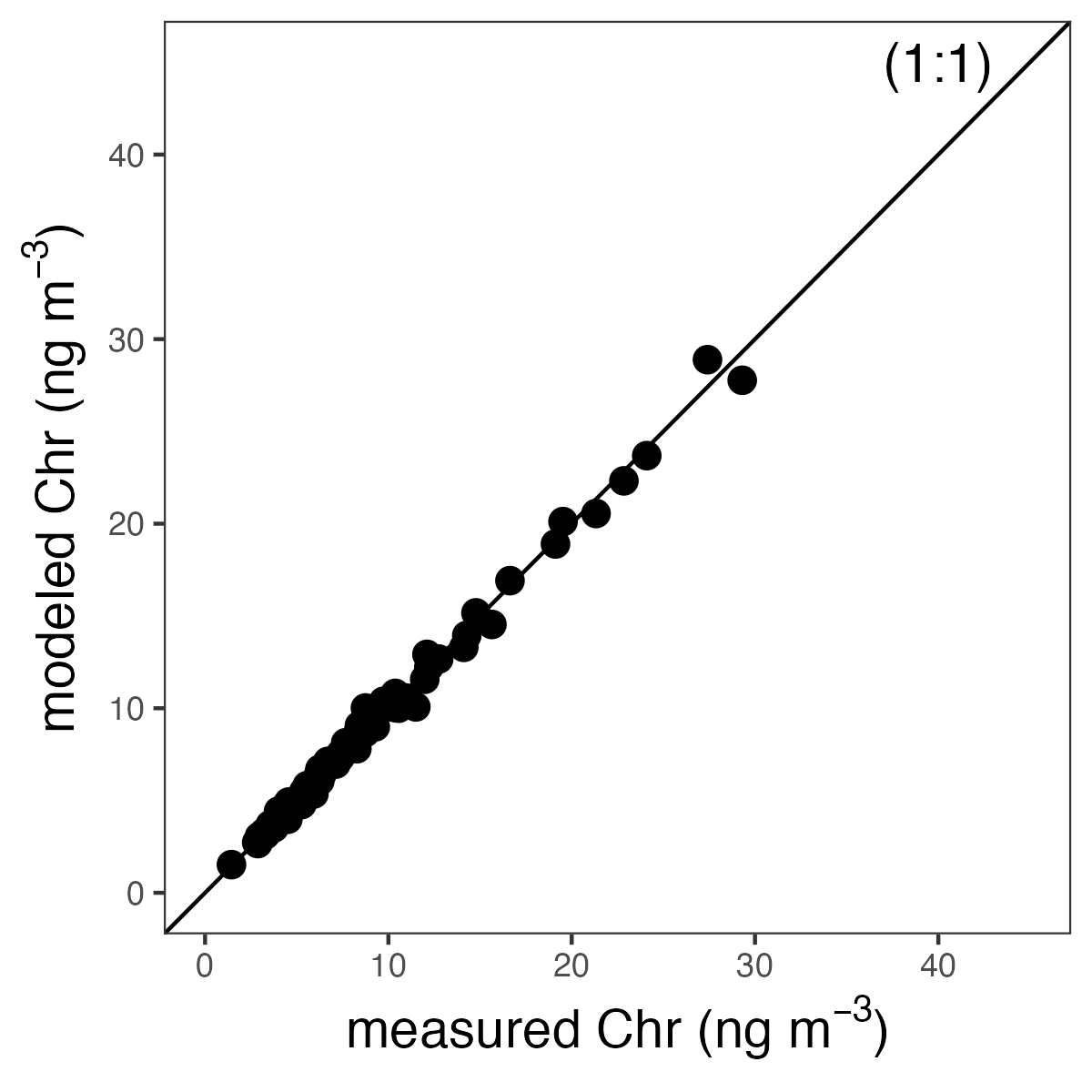

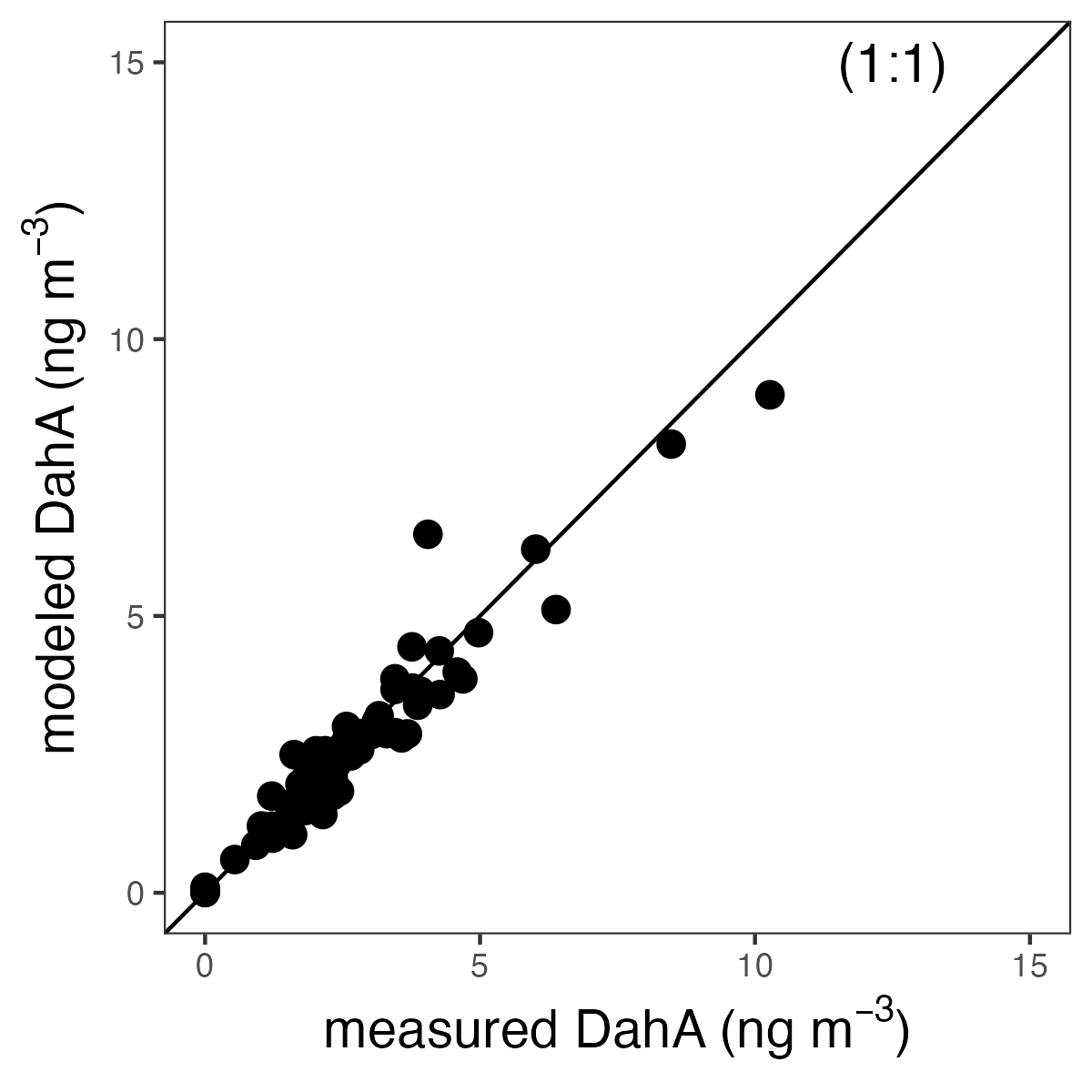

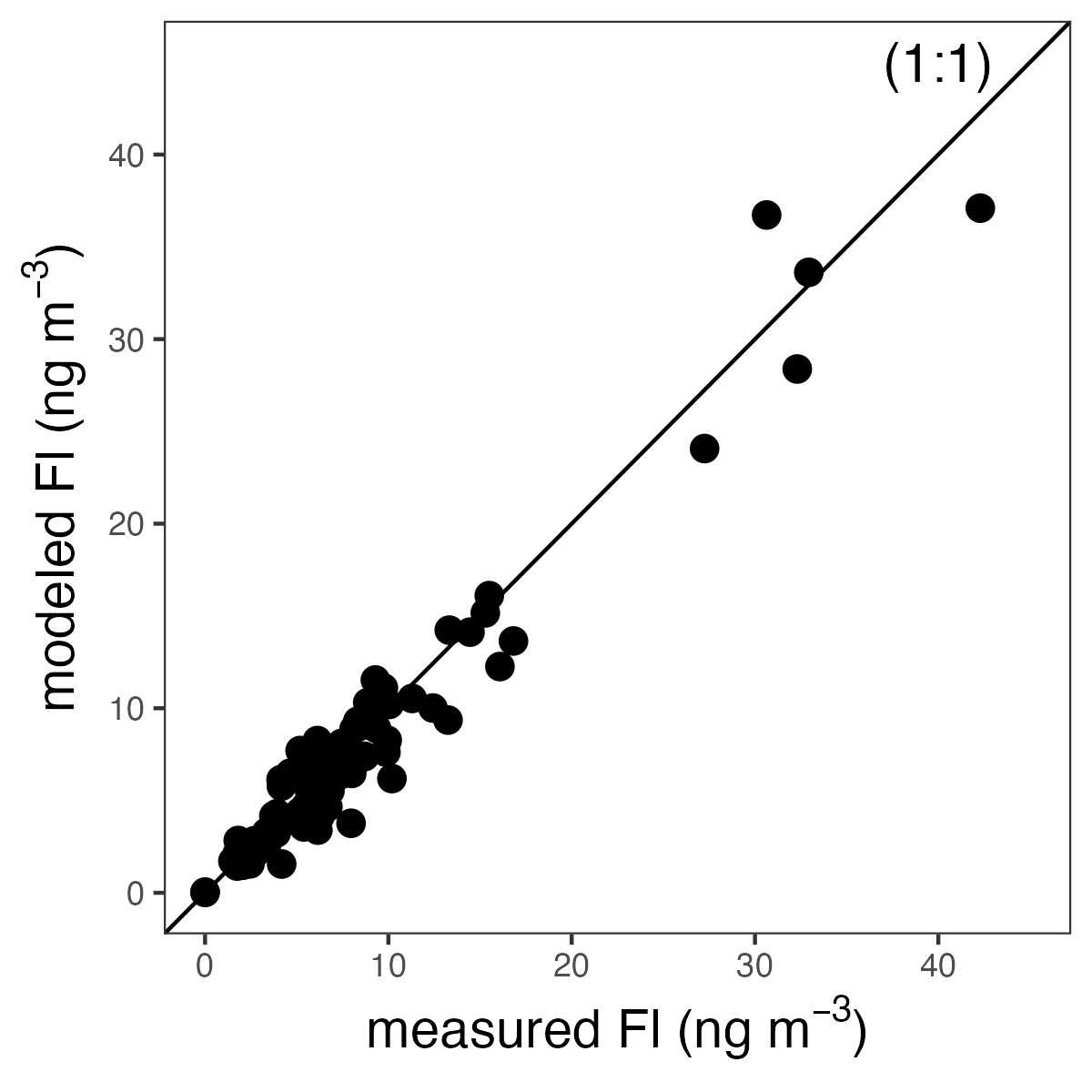

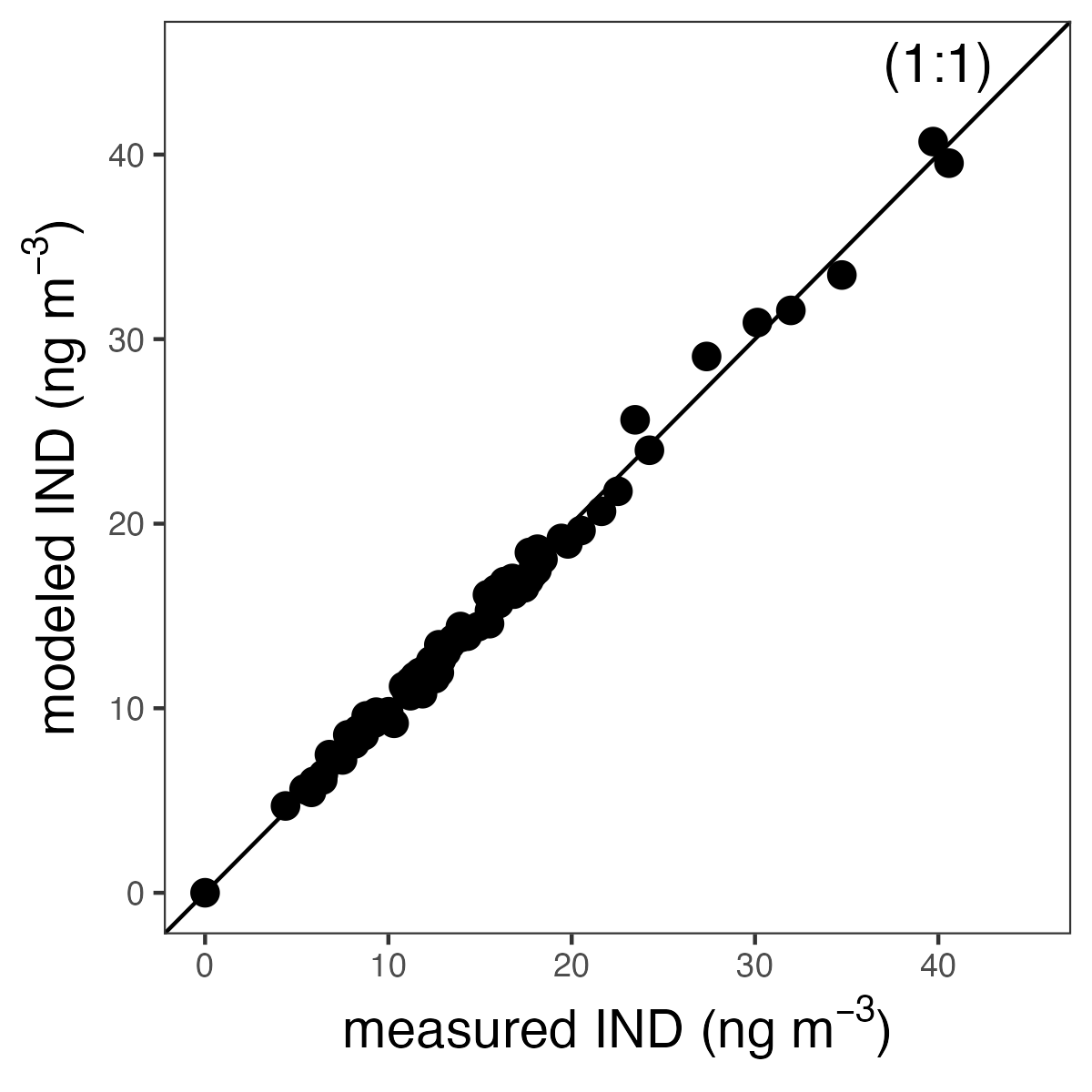

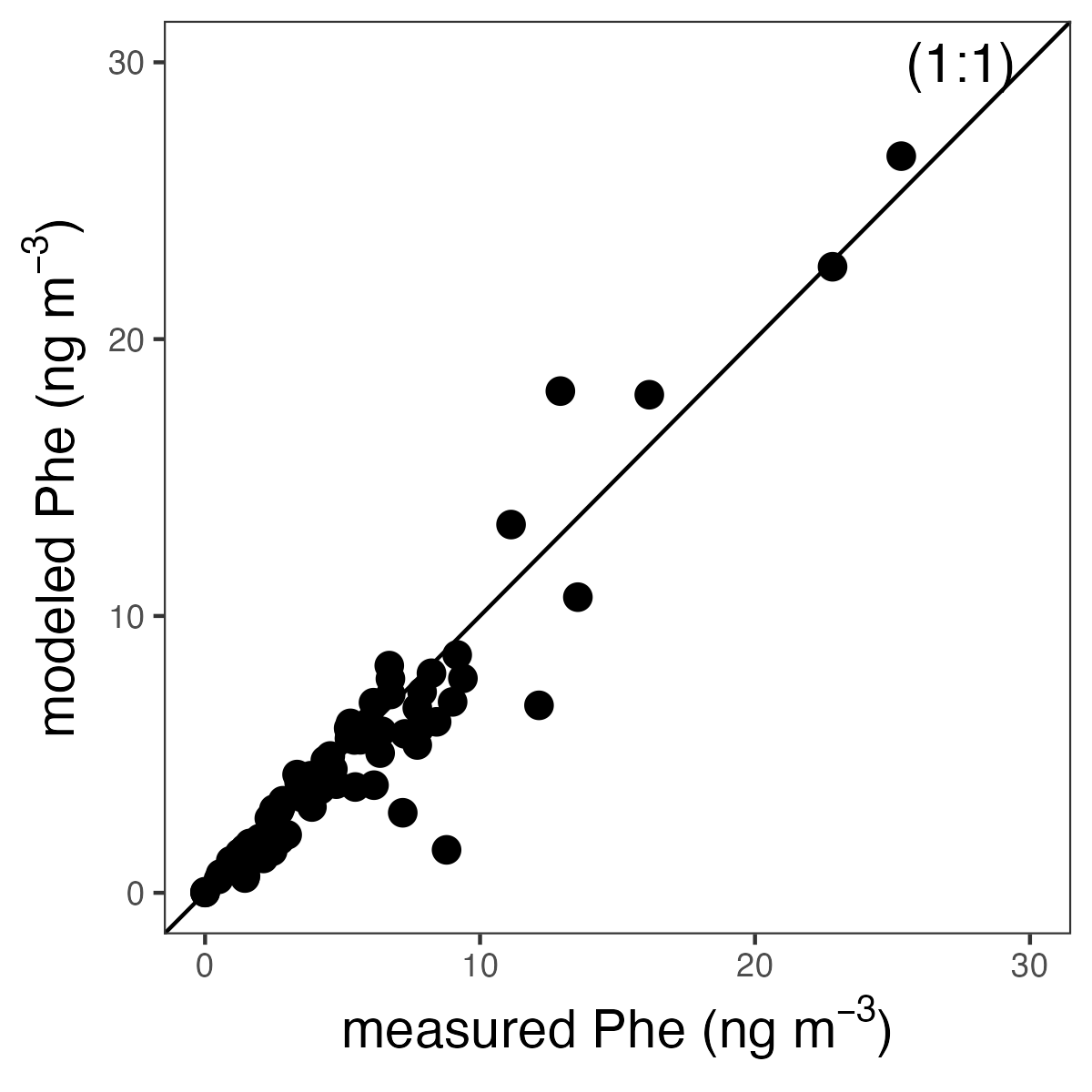

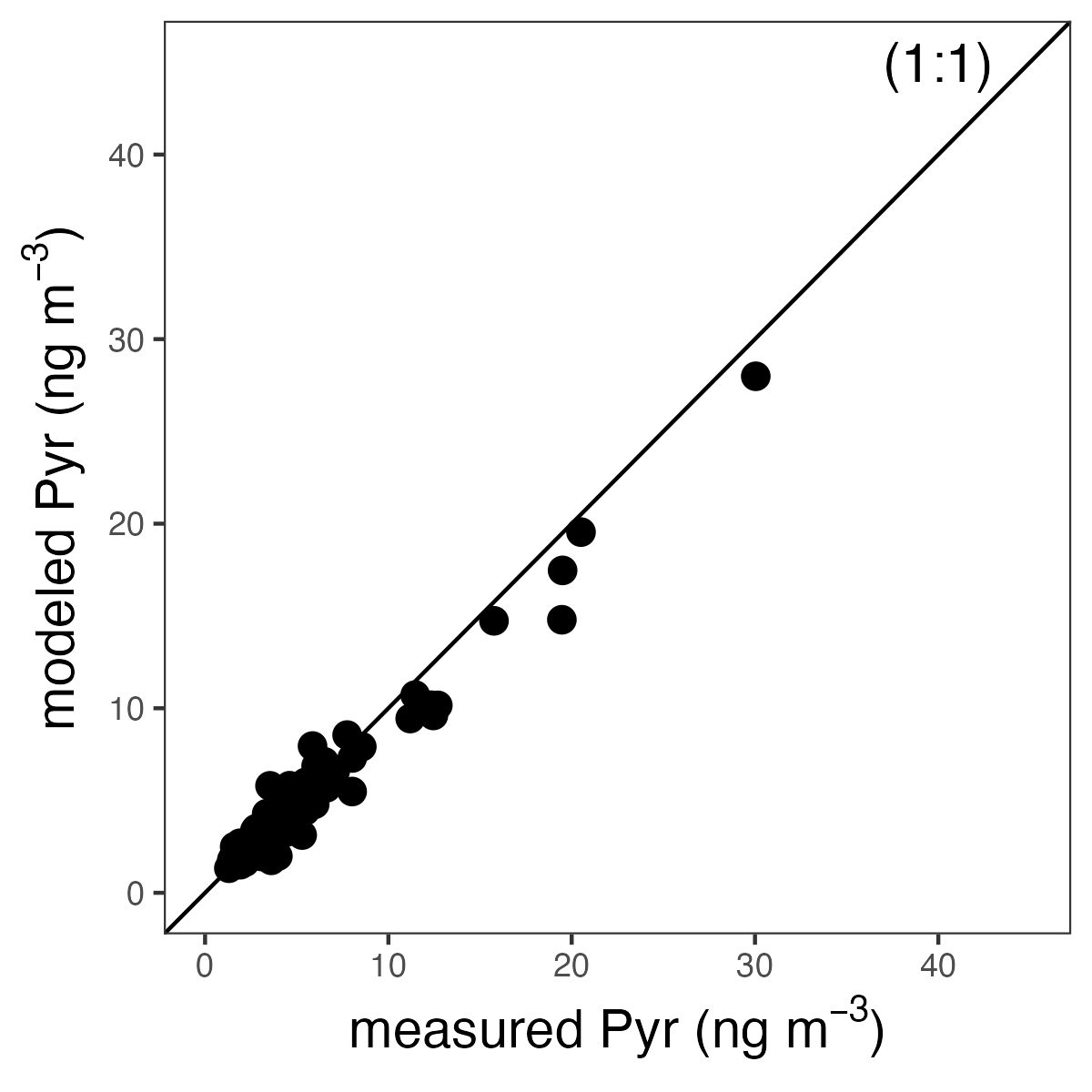

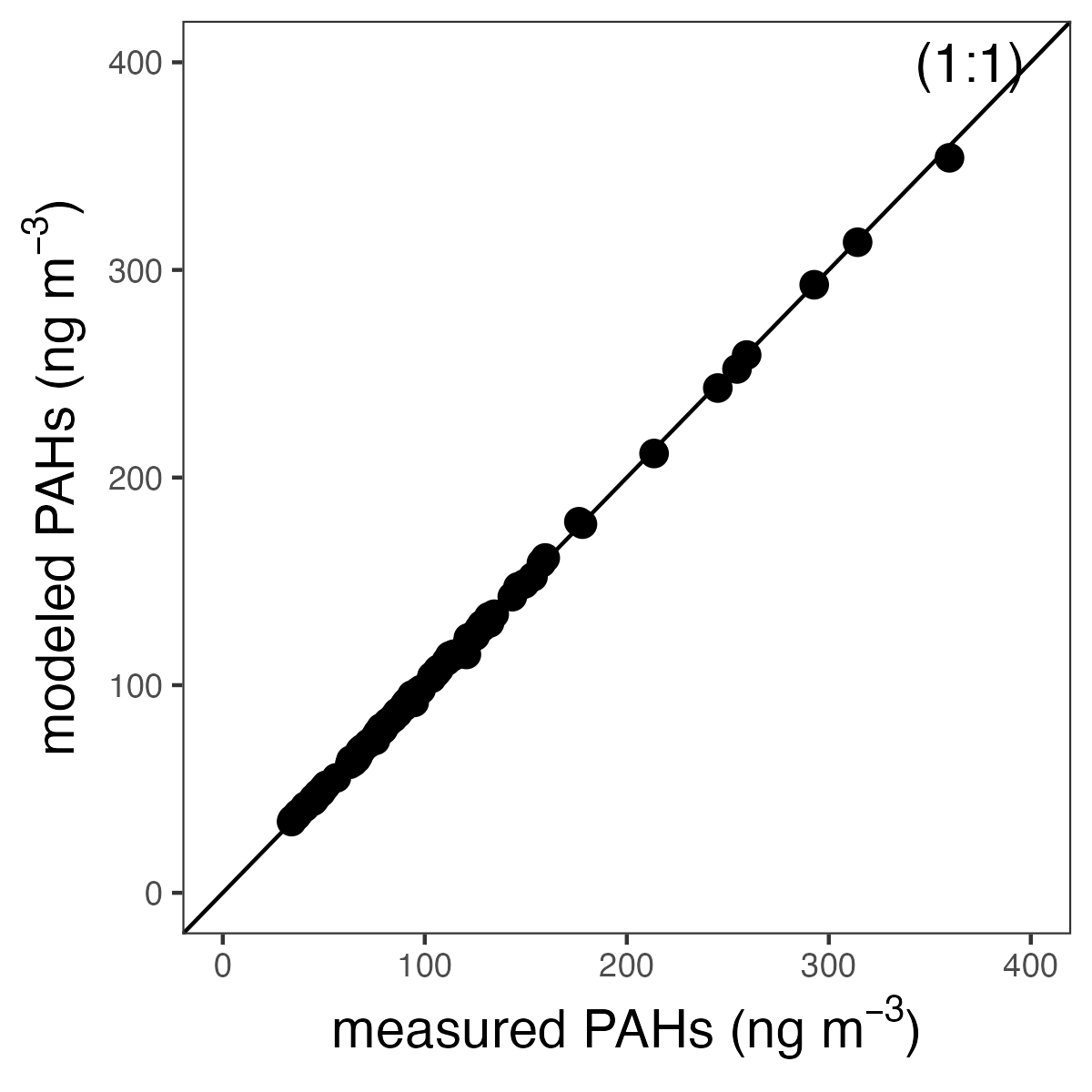
*

Figure S3 Comparisons between measured and modeled PAH individuals

# Figure S4 Associations between source-specific PAHs and cytokines, stratiﬁed by smoking status


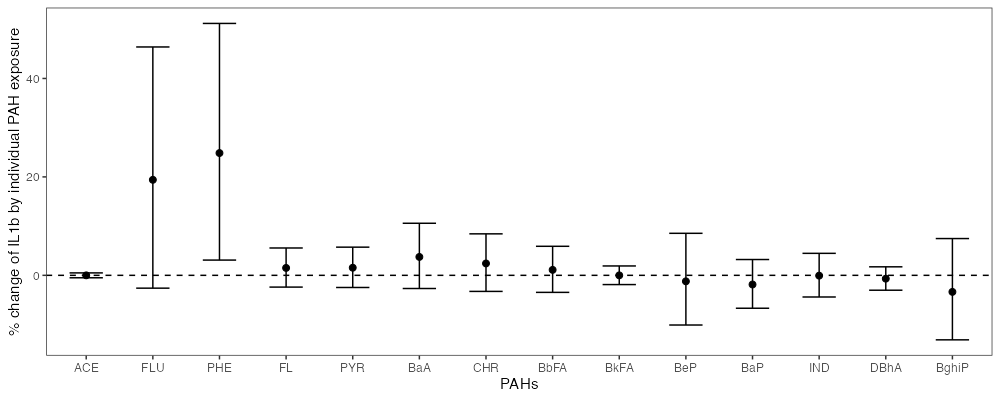


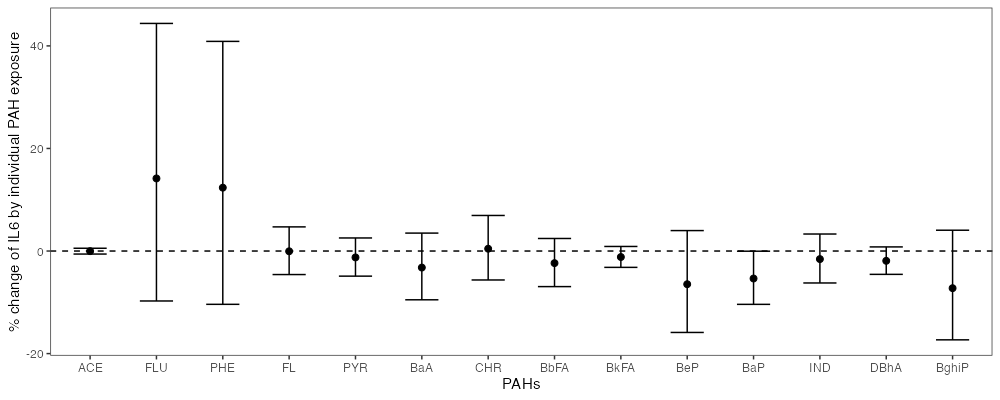

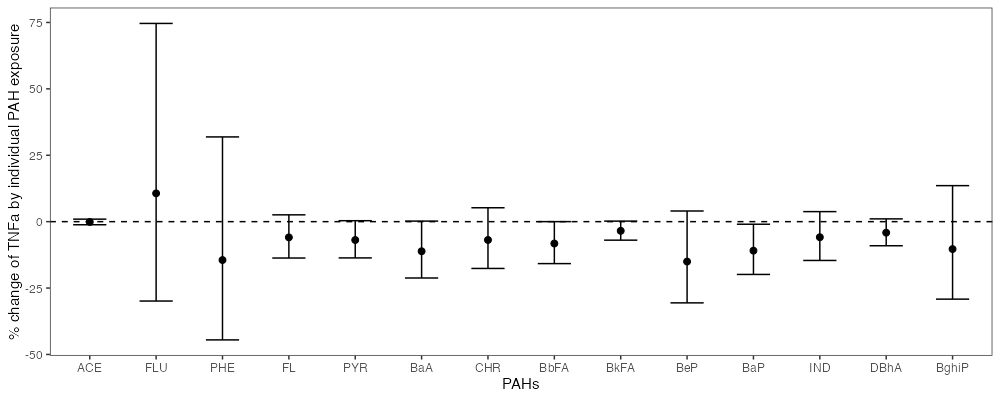


# Figure S5 Associations between source-specific PAHs and cytokines, stratiﬁed by smoking status









Figure S4 Associations between source-specific PAHs and cytokines, stratiﬁed by smoking status (F1: Cooking Fumes, F2: Diesel Vehicle Emission [DV], F3: Coal Combustion, F4: Environmental Tobacco Smoking, F5: Gasoline Vehicle Emission, F6: Biomass Burning).

Note: IL, interleukin; TNFα, tumor necrosis alpha; IQR, interquartile range

# Figure S6 The estimates with 95%CI of inflammatory cytokines with per IQR increase of source-specific PM_2.5_ concentrations before and after controlling for OC, disease conditions, PM_2.5_ mass concentration, residual PM_2.5_ mass, concentration, or residual total PAHs mass concentration


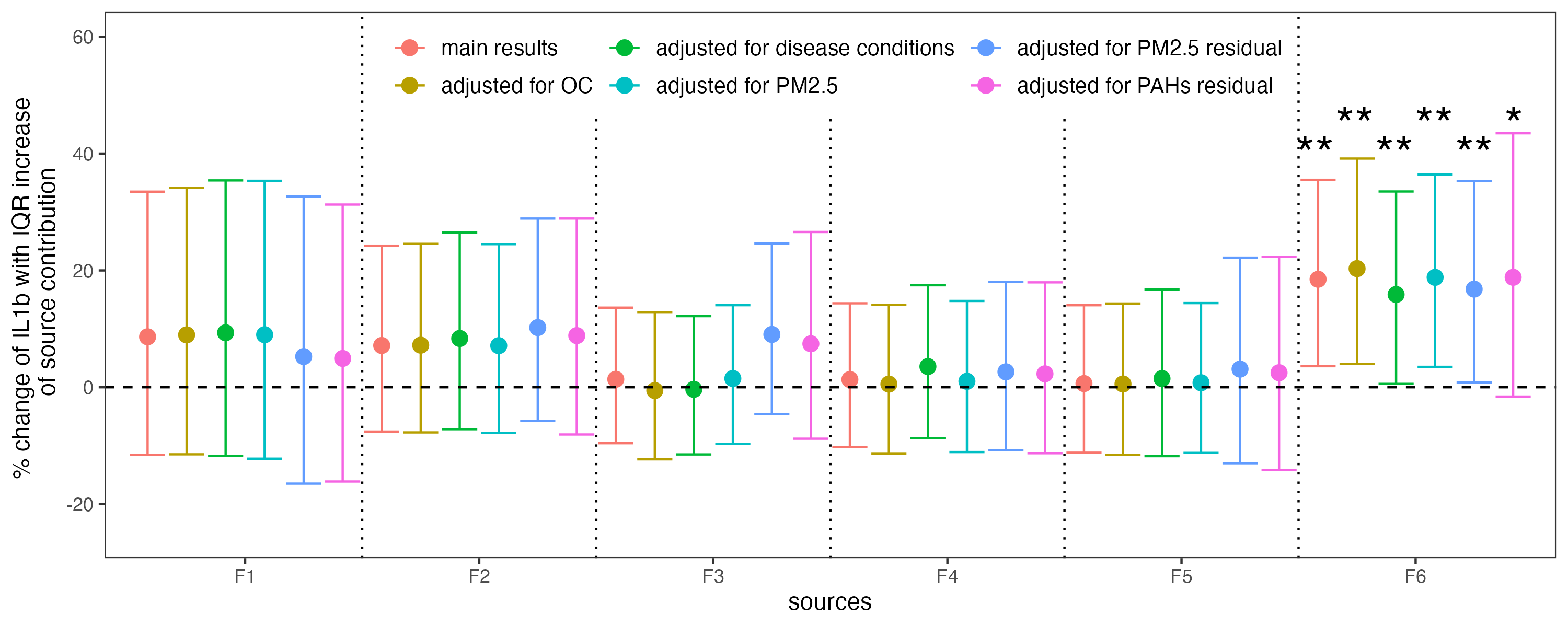


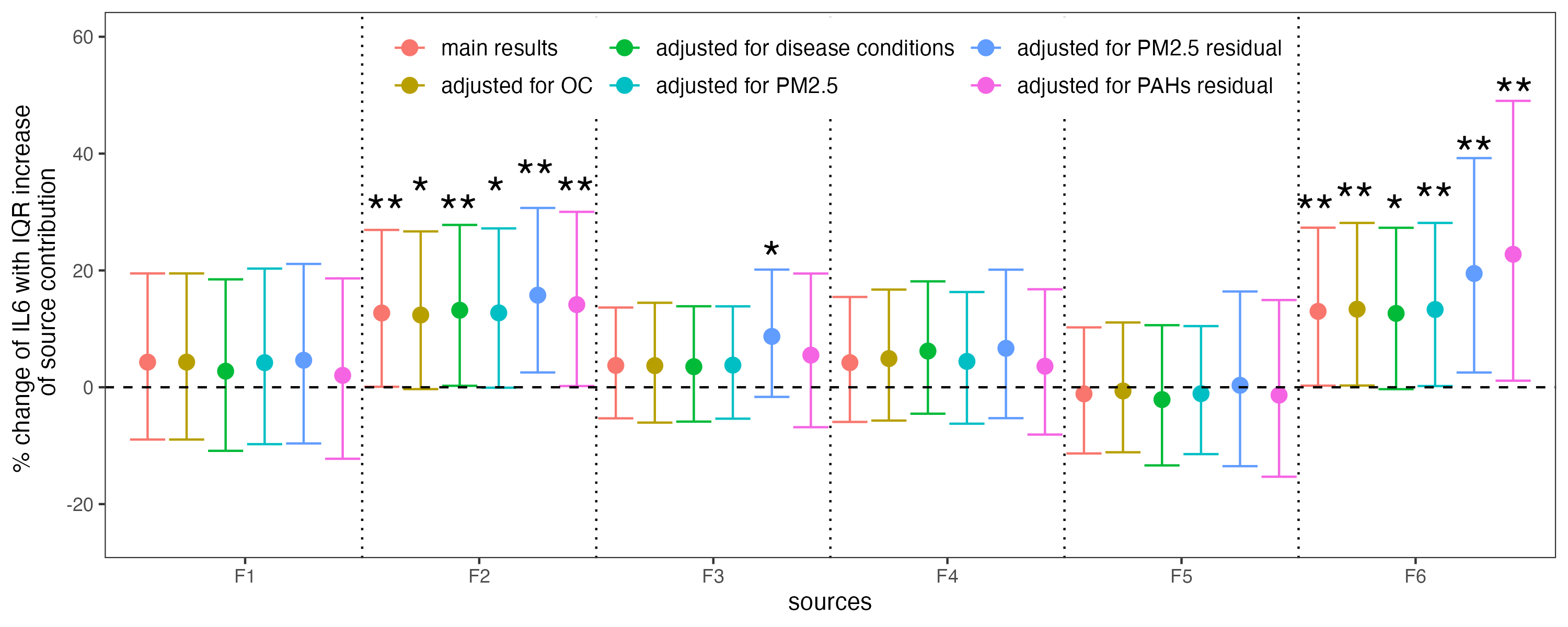


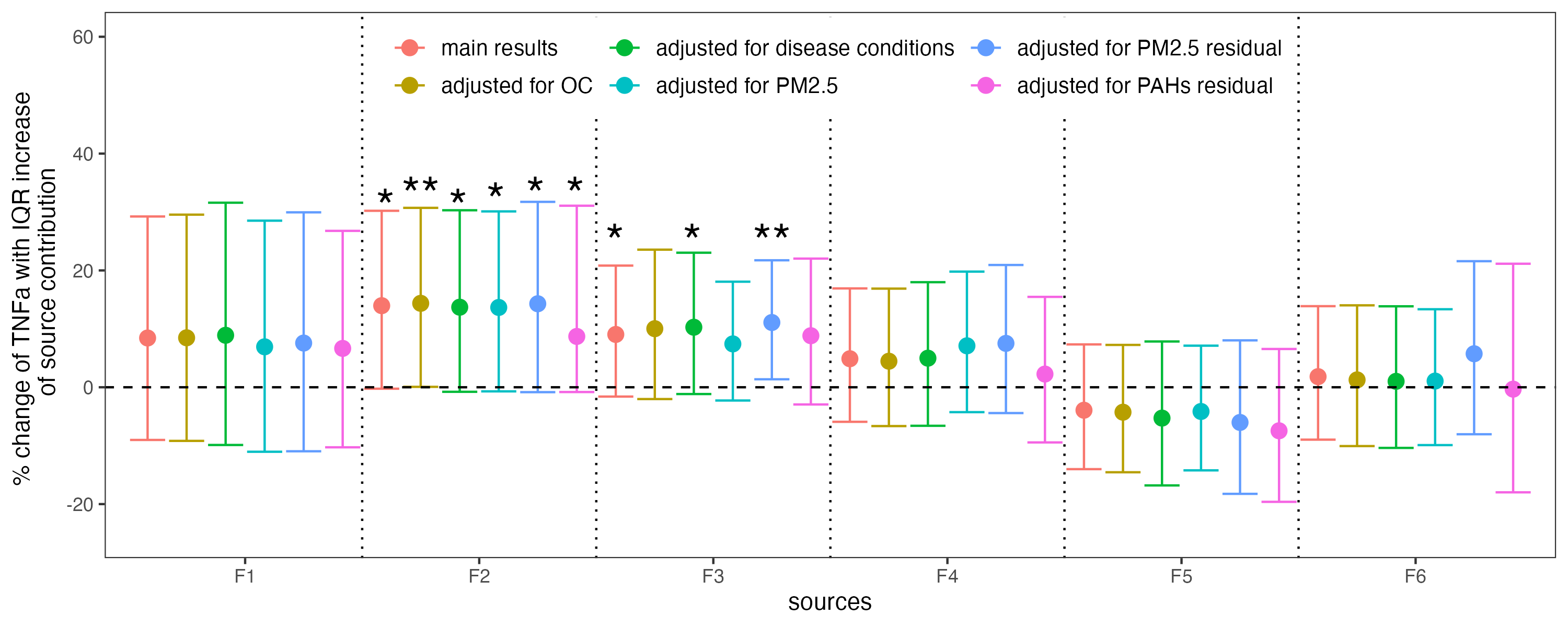


Figure S3 The estimates with 95%CI of inflammatory cytokines with per IQR increase of source-specific PM_2.5_ concentrations before and after controlling for OC, disease conditions, PM_2.5_ mass concentration, residual PM_2.5_ mass, concentration, or residual total PAHs mass concentration

(F1: Cooking Fumes, F2: Diesel Vehicle Emission, F3: Coal Combustion, F4: Environmental Tobacco Smoking, F5: Gasoline Vehicle Emission, F6: Biomass Burning).

Note: CI, confidence interval; IL, interleukin; TNFα, tumor necrosis alpha; IQR, interquartile range; * 0.05 < P value < 0.1; ** P value <0.05.

Note: ** P value < 0.05; * P value < 0.1; CI, confidence interval; IL, interleukin;

# Table S1 Summary of the PAH analysis and Quality Control/Quality Assurance

| PAH | Number  of rings | Ion  monitored | Surrogate  Standard | Internal  Standard | Method Detection Limit (ng/ml) | Recovery % | RSD , % | percentage below the LODs, % |
| --- | --- | --- | --- | --- | --- | --- | --- | --- |
| FLU | 3 | 166 | D_10_-Fluorene | D_10_-Acenaphthene | 6 | 86.61 | 7.71 | 1.6% |
| PHE | 3 | 178 | D_10_-Fluoranthene | D_10_-Phenanthrene | 8 | 89.60 | 3.78 | 2.6% |
| PYR | 4 | 202 | D_10_-Fluoranthene | D_12_-Chrysene | 6 | 84.54 | 5.61 | 5.2% |
| BaA | 4 | 216 | D_10_-Fluoranthene | D_12_-Chrysene | 10 | 81.22 | 6.78 | 2.1% |
| BbF | 4 | 216 | D_10_-Fluoranthene | D_10_-Acenaphthene | 10 | 84.91 | 9.25 | 6.8% |
| CHR | 4 | 228 | D_10_-Fluoranthene | D_12_-Chrysene | 10 | 84.91 | 7.14 | 5.1% |
| BkF | 5 | 252 | D_12_-Benzo(a)pyrene | D_12_-Perylene | 10 | 87.08 | 9.63 | 5.7% |
| BeP | 5 | 252 | D_12_-Benzo(a)pyrene | D_12_-Perylene | 12 | 88.83 | 4.26 | 6.3% |
| BaP | 5 | 252 | D_12_-Benzo(a)pyrene | D_12_-Perylene | 12 | 92.68 | 6.79 | 4.0% |
| IND | 6 | 276 | D_12_-Benzo(a)pyrene | D_10_-Acenaphthene | 10 | 81.15 | 12.74 | 7.2% |
| BghiP | 6 | 276 | D_12_-Benzo(a)pyrene | D_10_-Acenaphthene | 10 | 82.48 | 14.52 | 7.5% |
| COR | 7 | 300 | D_12_-Benzo(a)pyrene | D_10_-Acenaphthene | 12 | 86.90 | 6.80 | 6.0% |

# Table S2 PAHs concentration in standard solution grads (μg/ml)

| PAHs | Original solution | PAHs 1#  1/100 | PAHs 2#  1/200 | PAHs 3#  1/385 | PAHs 4#  1/500 | PAHs 5#  1/1250 |
| --- | --- | --- | --- | --- | --- | --- |
| FLU | 200.2 | 2 | 1 | 0.52 | 0.4 | 0.32 |
| PHE | 99.9 | 1 | 0.5 | 0.26 | 0.2 | 0.16 |
| PYR | 100.1 | 1 | 0.5 | 0.26 | 0.2 | 0.16 |
| BaA | 100.1 | 1 | 0.5 | 0.26 | 0.2 | 0.16 |
| CHR | 100.2 | 1 | 0.5 | 0.26 | 0.2 | 0.16 |
| BbF | 200.2 | 2 | 1 | 0.52 | 0.4 | 0.32 |
| BkF | 100.2 | 1 | 0.5 | 0.26 | 0.2 | 0.16 |
| BeP | 102 | 1.02 | 0.51 | 0.27 | 0.2 | 0.16 |
| BaP | 100 | 1 | 0.5 | 0.26 | 0.2 | 0.16 |
| IND | 100 | 1 | 0.5 | 0.26 | 0.2 | 0.16 |
| BghiP | 200.1 | 2 | 1 | 0.52 | 0.4 | 0.32 |
| COR | 100 | 1 | 0.5 | 0.26 | 0.2 | 0.16 |

# Table S3 Basic characteristics and clinical parameters of participants (N=82)

| Variable | Median (5^th^, 95^th^ percentiles) or percent |
| --- | --- |
| Hypertension | 44.6% |
| Hyperlipidemia | 33.0% |
| Diabetes | 17.0% |
| SBP (mmHg) | 138.5 (120,160) |
| DBP (mmHg) | 86 (70,117) |
| LDL (mmol/L) | 3.25 (2.17,4.42) |
| HDL (mmol/L) | 1.28 (0.89,1.75) |
| Triglycerides (mmol/L) | 1.41 (0.66,3.02) |
| Total cholesterol (mmol/L) | 5.43 (4.09,7.04) |
| Fast glucose (mmol/L) | 5.43 (4.72,8.60) |

Note: SBP, systolic blood pressure; DBP, diastolic blood pressure; LDL, low-density lipoprotein; HDL, high-density lipoprotein.

# Table S4 Estimated changes of cytokines with IQR increase of source-specific PAHs concentrations analyzed by PMF exposure stratiﬁed by smoking status.

| Sources | IL1β | | |  | IL6 | | |  | TNFα | | |
| --- | --- | --- | --- | --- | --- | --- | --- | --- | --- | --- | --- |
|  | Smoker |  | Non-smoker |  | Smoker |  | Non-smoker |  | Smoker |  | Non-smoker |
| Cooking Fumes | 2.8  (-29.0,48.8) |  | 12.6  (-13.4,46.4) |  | 13.1  (-19.6,59.1) |  | 2.1  (-13.0,19.7) |  | 17.3  (-10.2,53.2) |  | 3.3  (-18.0,30.0) |
| Diesel Vehicle Emission | -1.5  (-39.1,59.4) |  | 8.8  (-7.6,28.2) |  | -5.6  (-33.3,33.7) |  | 10.5  (-1.1,23.6) **^*^** |  | 9.2  (-24.5,57.9) |  | 14.9  (-1.1,33.6) **^*^** |
| Coal  Combustion | -6.1  (-22.7,14.0) |  | 6.2  (-11.1,26.8) |  | -1.0  (-18.8,20.7) |  | 5.5  (-6.2,18.7) |  | 10.3  (-6.8,30.7) |  | 10.0  (-6.7,29.7) |
| Environmental Tobacco Smoking | -1.8  (-27.0,32.1) |  | 1.2  (-10.2,14.1) |  | -5.7  (-29.6,26.3) |  | 5.5  (-3.9,15.9) |  | -13.9  (-33.0,10.7) |  | 8.2  (-2.4,19.8) |
| Gasoline Vehicle Emission | 5.4  (-22.3,43.0) |  | -1.4  (-17.6,18.0) |  | 1.2  (-25.5,37.4) |  | -2.3  (-15.9,13.4) |  | -3.1  (-25.4,25.8) |  | -5.7  (-19.8,10.9) |
| Biomass  Burning | 12.8  (-13.4,47.0) |  | **22.7**  **(3.1,45.9) ^**^** |  | 8.1  (-13.0,34.2) |  | **19.5**  **(3.3,38.1) ^**^** |  | 3.4  (-19.6,33.0) |  | 1.2  (-11.2,15.4) |

Note: * 0.05 < P value < 0.1; ** P value <0.05

# Table S5 The source apportionment results in the study city at different years

| Study Period | Fugitive  Dust | Secondary  source | Coal  Combustion | Biomass  Burning | Vehicle  emission | Industrial  emission | Reference |
| --- | --- | --- | --- | --- | --- | --- | --- |
| Winter study | | | | | | | |
| Dec. 2011 ^#^ | - | - | 23.7 | 22.7 | 27.0 (diesel)  26.6 (gasoline) | - | This study |
| Jan. to Apr., 2014 ^*^ | 17.0 | - | 29.3 | 12.7 | 41.0 | - | [*Wen et al.* [2018]](#_ENREF_10) |
| Dec. 2013  to Jan. 2014 ^&^ | 17.7 | 34.3 | 12.6 | - | 27.0 | - | [*Wu et al.* [2015]](#_ENREF_11) |
| Whole year study (the results of winter were not specified in the references) | | | | | | | |
| Feb. to  Mar., 2016 ^&^ | 8.6 | 31.7 | 23.6 | | 27.6 | 1.8 | [*Liu et al.* [2020]](#_ENREF_3) |
| Jan., 2017 ^&^ | 10.1 | 38.1 | 17.1 | 9.1 | 25.6 | - | [*Yuan et al.* [2018]](#_ENREF_13) |
| 2011 ^&^ | 22 | 36 | 30 | - | 12 | - | [[*Shi et al.*, 2016](#_ENREF_7)] |
| Jan. 2013 to Jan. 2014 ^&^ | 20.3 | 41.1 | 24.6 | | 14 | - | [*Tian et al.* [2016]](#_ENREF_8) |
| May 2013 to Jan. 2014 ^&^ | 19 | 41 | 21 | | 17 | - | [*Tian et al.* [2018]](#_ENREF_9) |
| 2014 ^&^ | 26.4 | 13.1 | 25.6 | | 23.5 | 11.4 | [*Gao et al.* [2018]](#_ENREF_1) |
| Jun. 2014 to Apr. 2015 ^&^ | 11.7 | 29.2 | 12.4 | 5.3 | 15.2 | 11.7 | [*Huang et al.* [2017]](#_ENREF_2) |
| Apr. 2014 to Jan. 2015 ^&^ | 21.3 | 39 | 18 | - | 17 | - | [*Shi et al.* [2018]](#_ENREF_6) |
| Feb. to Oct., 2016 ^&^ | 10.9 | 38.1 | 17.3 | | 23.5 | 4 | [*Liu et al.* [2020]](#_ENREF_3) |
| Oct. 2017 to Aug. 2018 ^&^ | 7 | 30 | 25 | 2 | 21 | 5 | [*Zhang et al.* [2021]](#_ENREF_14) |
| 2017-2019 ^&^ | 14.2 | 33.5 | 19.2 (including industrial emission) | 4.6 | 19.8 | - | [*Xiao et al.* [2020]](#_ENREF_12) |

^#.^ Target chemical compositions: PM_2.5_ bound PAHs (personal exposure sample)

^*.^ Target chemical compositions: PM_2.5_ WSOC (Ambient sample)

^&.^ Target chemical compositions: PM_2.5_ bound elements, water-soluble ions and carbonaceous fractions (Ambient sample)

# Reference

Gao, J., K. Wang, Y. Wang, S. Liu, C. Zhu, J. Hao, H. Liu, S. Hua, and H. Tian (2018), Temporal-spatial characteristics and source apportionment of PM2.5 as well as its associated chemical species in the Beijing-Tianjin-Hebei region of China, *Environmental Pollution*, *233*, 714-724, doi:10.1016/j.envpol.2017.10.123.

Huang, X., Z. Liu, J. Liu, B. Hu, T. Wen, G. Tang, J. Zhang, F. Wu, D. Ji, L. Wang, and Y. Wang (2017), Chemical characterization and source identification of PM2.5 at multiple sites in the Beijing–Tianjin–Hebei region, China, *Atmos. Chem. Phys.*, *17*(21), 12941-12962, doi:10.5194/acp-17-12941-2017.

Liu, B., Y. Li, L. Wang, X. Bi, H. Dong, X. Sun, Z. Xiao, Y. Zhang, and Y. Feng (2020), Source directional apportionment of ambient PM2.5 in urban and industrial sites at a megacity in China, *Atmospheric Research*, *235*, 104764, doi:10.1016/j.atmosres.2019.104764.

Paatero, P. (1997), Least squares formulation of robust non-negative factor analysis, *Chemometrics and Intelligent Laboratory Systems*, *37*(1), 23-35, doi:10.1016/S0169-7439(96)00044-5.

Paatero, P., and U. Tapper (1994), Positive matrix factorization: A non-negative factor model with optimal utilization of error estimates of data values, *Environmetrics*, *5*(2), 111-126, doi:10.1002/env.3170050203.

Shi, G., J. Liu, H. Wang, Y. Tian, J. Wen, X. Shi, Y. Feng, C. E. Ivey, and A. G. Russell (2018), Source apportionment for fine particulate matter in a Chinese city using an improved gas-constrained method and comparison with multiple receptor models, *Environmental Pollution*, *233*, 1058-1067, doi:10.1016/j.envpol.2017.10.007.

Shi, G., J. Xu, X. Peng, Y.-Z. Tian, W. Wang, B. Han, Y. Zhang, Y.-C. Feng, and A. G. Russell (2016), Using a new WALSPMF model to quantify the source contributions to PM2.5 at a harbour site in China, *Atmospheric Environment*, *126*, 66-75, doi:10.1016/j.atmosenv.2015.11.046.

Tian, Y., G. Chen, H. Wang, Y. Huang-Fu, G. Shi, B. Han, and Y. Feng (2016), Source regional contributions to PM2.5 in a megacity in China using an advanced source regional apportionment method, *Chemosphere*, *147*, 256-263, doi:10.1016/j.chemosphere.2015.12.132.

Tian, Y., J. Liu, S. Han, X. Shi, G. Shi, H. Xu, H. Yu, Y. Zhang, Y. Feng, and A. G. Russell (2018), Spatial, seasonal and diurnal patterns in physicochemical characteristics and sources of PM2.5 in both inland and coastal regions within a megacity in China, *Journal of Hazardous Materials*, *342*, 139-149, doi:10.1016/j.jhazmat.2017.08.015.

Wen, J., G. Shi, Y. Tian, G. Chen, J. Liu, Y. Huang-Fu, C. E. Ivey, and Y. Feng (2018), Source contributions to water-soluble organic carbon and water-insoluble organic carbon in PM2.5 during Spring Festival, heating and non-heating seasons, *Ecotoxicology and Environmental Safety*, *164*, 172-180, doi:10.1016/j.ecoenv.2018.08.002.

Wu, H., Y.-f. Zhang, S.-q. Han, J.-h. Wu, X.-h. Bi, G.-l. Shi, J. Wang, Q. Yao, Z.-y. Cai, J.-l. Liu, and Y.-c. Feng (2015), Vertical characteristics of PM2.5 during the heating season in Tianjin, China, *Science of The Total Environment*, *523*, 152-160, doi:10.1016/j.scitotenv.2015.03.119.

Xiao, Z., H. Xu, L. Li, P. Li, J. Yuan, M. Tang, N. Yang, N. Zheng, and K. CHen (2020), Characterization and Source Apportionment of PM2.5 Based on the Online Observation in Tianjin (In Chinese with English Abstract), *Environmental Science*, *41*(10), 4355-4362, doi:10.13227/j.hjkx.202001185.

Yuan, J., B. Liu, Y. Cheng, Z. Xiao, H. XU, and Y. Guan (2018), Study on characteristics of PM2.5 and chemical components and source apportionment of high temporal resolution in January 2017 in Tianjin urban area (In Chinese with English Abstract), *Acta Scientiae Circumstantiae*, *38*(3), 1090-1101, doi:10.13671/j.hjkxxb.2017.0435.

Zhang, W., X. Peng, X. Bi, Y. Cheng, D. Liang, J. Wu, Y. Tian, Y. Zhang, and Y. Feng (2021), Source apportionment of PM2.5 using online and offline measurements of chemical components in Tianjin, China, *Atmospheric Environment*, *244*, 117942, doi:10.1016/j.atmosenv.2020.117942.
